# Advanced Composites Inspired by Biological Structures and Functions in Nature: Architecture Design, Strengthening Mechanisms, and Mechanical‐Functional Responses

**DOI:** 10.1002/advs.202207192

**Published:** 2023-03-19

**Authors:** Hanqing Dai, Wenqing Dai, Zhe Hu, Wanlu Zhang, Guoqi Zhang, Ruiqian Guo

**Affiliations:** ^1^ Academy for Engineering and Technology Institute for Electric Light Sources Fudan University Shanghai 200433 China; ^2^ School of Materials Science and Engineering Shanghai Jiao Tong University Shanghai 200240 China; ^3^ School of Information Science and Technology Fudan University Shanghai 200433 China; ^4^ Department of Microelectronics Delft University of Technology Delft CD 2628 Netherlands

**Keywords:** architecture, bionics, composites, structure properties

## Abstract

The natural design and coupling of biological structures are the root of realizing the high strength, toughness, and unique functional properties of biomaterials. Advanced architecture design is applied to many materials, including metal materials, inorganic nonmetallic materials, polymer materials, and so on. To improve the performance of advanced materials, the designed architecture can be enhanced by bionics of biological structure, optimization of structural parameters, and coupling of multiple types of structures. Herein, the progress of structural materials is reviewed, the strengthening mechanisms of different types of structures are highlighted, and the impact of architecture design on the performance of advanced materials is discussed. Architecture design can improve the properties of materials at the micro level, such as mechanical, electrical, and thermal conductivity. The synergistic effect of structure makes traditional materials move toward advanced functional materials, thus enriching the macroproperties of materials. Finally, the challenges and opportunities of structural innovation of advanced materials in improving material properties are discussed.

## Graphical abstract

In this work, the progress of structural materials is reviewed, the strengthening mechanisms of different types of structures are highlighted, and the impact of architecture design on the performance of advanced materials is discussed. Finally, the challenges and opportunities of structural innovation of advanced materials in improving material properties are discussed.

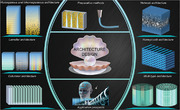

## 1 Introduction

Advanced materials through architecture design exhibit excellent mechanical properties, functional physical properties, and unique coupling properties.^[^ ^1^, ^2^, ^3^, ^4^, ^5^ ^]^ They play a fundamental role in promoting the progress of materials science, biology, and physics.^[^ ^6^, ^7^, ^8^, ^9^, ^10^ ^]^ Although many microstructures have been researched, designed, and applied to improve the performance of materials and gained beautiful achievements, the design and development of microstructure architectures are far more than this.^[^ ^11^, ^12^, ^13^, ^14^, ^15^ ^]^ The existing architectures of nature and the infinite imaginations of human beings are the sources of promoting the architecture design of advanced materials. These microstructures are very attractive for the subversive improvement of material properties, but the study of their strengthening mechanism and interaction properties still faces many challenges.

The evolution of human civilization has always been accompanied by the design and development of material structures. Since ancient times, structural materials with special functions have been mainly used in the fields of bridges, houses, vehicles, and industrial manufacturing.^[^ ^16^, ^17^, ^18^, ^19^, ^20^ ^]^ In the early stage, due to the low complexity of structural materials, the large size of components, and the immature preparation technology, the performance improvement of composite materials is limited. In the past few decades, due to the limitation of synthesis and control methods, most of the applied research focused on simple structural materials. These structural materials with lightweight and high mechanical properties are mainly designed and produced from the perspective of basic mechanics. Generally, these studies are mainly about the uniform distribution of reinforcement, interface modification, mixing ratio, size parameters, and strengthening mechanism.^[^ ^21^, ^22^, ^23^, ^24^, ^25^, ^26^ ^]^ Since the last decade, many excellent composites have been produced by imitating the structure of natural materials.^[^ ^27^, ^28^, ^29^, ^30^ ^]^ The design and preparation of biomimetic composites usually need to scale up the size of biological structure, which is conducive to its large‐scale synthesis and processing in the industry. However, the maximization of the properties and the diversity of the functions of the composites need to improve the fineness of the preparation technology and the complexity of the microstructure architecture. With the further development of characterization, simulation, modeling, and manufacturing techniques, scientists increasingly believe that bio‐inspired structural materials with high mechanical properties and functionality comparable to natural materials can be produced.

**Figure 1:**
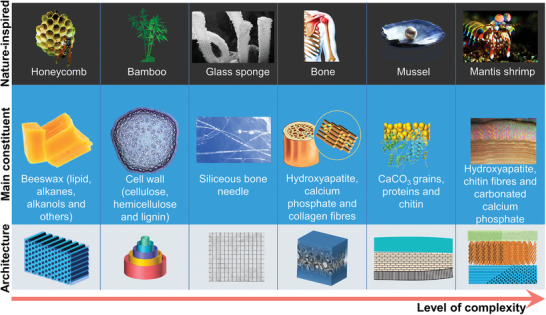
Typical architectures of nature‐inspired. The compositions and unique structures across multiple length scales of biomaterials are key to achieving extraordinary mechanical properties and functionality. Different species of organisms have structures of varying complexity, largely depending on the living environment and functional requirements. Inspired by these biological structures, a range of bionic architectures have been designed from simple to complex.

In recent years, the research of material microstructure architecture has made rapid progress. By imitating the microstructure in nature, some advanced materials with excellent performance have been designed.^[^ ^31^, ^32^, ^33^, ^34^, ^35^ ^]^ For example, some typical natural structures (honeycomb, bamboo, sponge, bone, pearl mussel, mantis shrimp) have excellent mechanical properties and have received extensive attention in bionics (**Figure** **1**).^[^ ^36^, ^37^, ^38^, ^39^, ^40^, ^41^, ^42^, ^43^, ^44^, ^45^ ^]^ Natural honeycombs are made of beeswax, which is mainly composed of lipids, alkanes, alkanols, and others.^[^ ^46^, ^47^ ^]^ These organic matters provide the honeycomb with basic hardness and toughness. Besides, the hexagonal cell of the honeycomb has the characteristics of lightweight, high strength, and strong energy absorption capacity.^[^ ^48^, ^49^, ^50^, ^51^ ^]^ Given these advantages, bionic honeycomb materials are developing rapidly in the direction of composite structures, multiple sizes, and integrated functions. The structure of natural bamboo is somewhat similar to that of honeycombs, but not identical. Bamboo is composed of cellulose, hemicellulose, and lignin.^[^ ^52^, ^53^ ^]^ The parenchyma cells of different lengths constitute fiber bundles. The thickness of the fiber wall is a gradient from outside to inside. Therefore, the bionic materials based on bamboo inspiration have honeycomb, fiber bundle, and gradient structures.^[^ ^54^, ^55^, ^56^ ^]^ Glass sponge is a typical net‐like lattice architecture. The thick silicon bone needles are at right angles to each other and form a tridimensional lattice with the thin bone needles.^[^ ^40^, ^57^, ^58^, ^59^ ^]^ This lattice architecture has received great attention due to its excellent mechanical stability. However, the potential mechanical properties of the hierarchical and multiscale structure of the net‐like lattice architecture still need to be further explored.

Bones support the whole weight of the human body and play an important role in driving body movement. Therefore, the bone architecture needs to have sufficient strength and certain toughness. The network of interconnected bone scaffolds is composed of hydroxyapatite, calcium phosphate, and collagen fibers.^[^ ^60^, ^61^, ^62^, ^63^, ^64^ ^]^ The growth rule of the bone network structure is affected by weight bearing, which makes it have the characteristics of good stress dispersion, high strength, and high density.^[^ ^65^, ^66^, ^67^, ^68^, ^69^, ^70^ ^]^ It is also a challenge to develop bionic bone materials based on these characteristics. Bone‐inspired materials should have mechanical properties, microstructure and biological characteristics similar to bones. The shell of mussels is composed of CaCO_3_ particles, protein, and chitin, which is a multistructure composite architecture.^[^ ^71^, ^72^, ^73^, ^74^ ^]^ In addition to the outermost cuticle, the prismatic layer in the middle and the nacre layer in the inner layer are the main part of mechanical energy dissipation.^[^ ^75^, ^76^, ^77^ ^]^ Nacre is a typical layered structure, which can deflect cracks and prevent crack propagation, so as to provide toughness and dissipate mechanical energy.^[^ ^78^, ^79^, ^80^, ^81^, ^82^ ^]^ The architecture of the prismatic layer is used to resist the impact load and wear.^[^ ^83^, ^84^, ^85^, ^86^ ^]^ The mantis shrimp has a better composite architecture to compete with mussels for survival. The dactyl club of mantis shrimp is mainly composed of mineralized hydroxyapatite, mineralized chitin fiber, and carbonated calcium phosphate.^[^ ^87^, ^88^, ^89^ ^]^ The impact surface of the outermost layer is a closely packed structure composed of mineralized hydroxyapatite particles and organic matter.^[^ ^90^, ^91^ ^]^ The impact region of the middle layer is a spatial complex network structure composed of wavy chitin fibers and vertical fiber tubes.^[^ ^87^, ^92^, ^93^ ^]^ The periodic region of the inner layer is a Bouligand structure composed of spiral fiber laminates.^[^ ^94^, ^95^, ^96^, ^97^ ^]^ The periodic region has been shown to have good damage tolerance and impact resistance. Furthermore, the coupling of the three different structures enables the dactyl club of mantis shrimp to withstand an impact force of 1500N, mainly because the composite architecture can resist the initiation of microcracks, as well as deflection and twist cracks.^[^ ^92^, ^98^, ^99^, ^100^, ^101^, ^102^ ^]^ Therefore, the multitype composite architectures are not only the result of natural evolution, but also the direction that material scientists need to study in depth.

Unlike other types of materials, advanced architecture materials not only have excellent mechanical properties but also develop in an intelligent, functional and environment‐friendly direction. With the progress of preparation technology, structural materials are endowed with various functions and good mechanical properties, such as high strength and toughness composite, smart materials for sensing and self‐healing, functional materials for drug delivery and self‐cleaning, and so on.^[^ ^103^, ^104^, ^105^, ^106^, ^107^ ^]^ Additionally, this field is developing in a more complex and functional direction that seamlessly blends artificial materials and natural materials, such as biodegradable cellulose composites, hydrogel structural materials for cell culture, biomimetic materials with molecular precision, etc.^[^ ^108^, ^109^, ^110^, ^111^ ^]^

**Figure 2:**
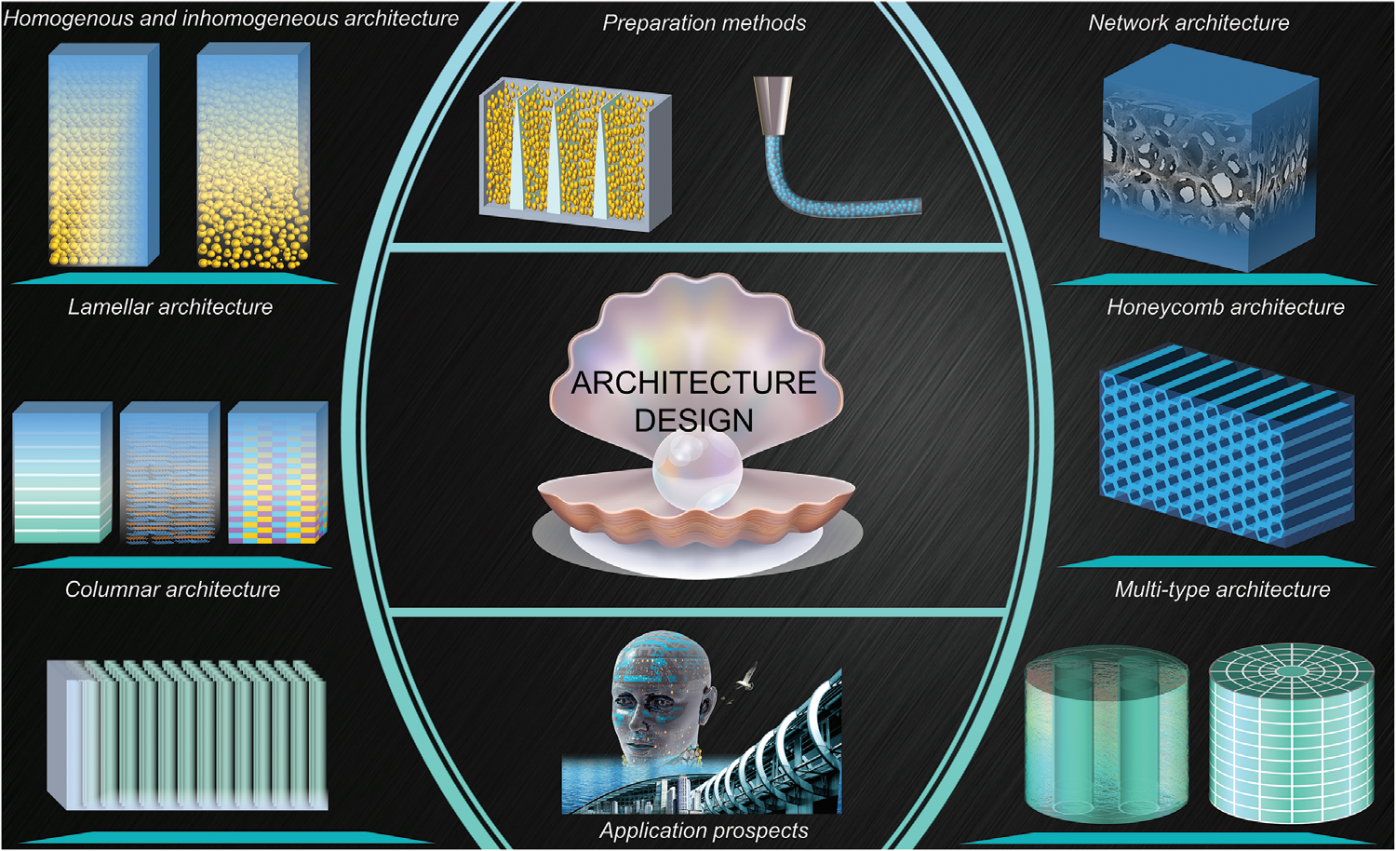
A schematic overview of the architecture, preparation, and application of advanced materials. Advanced biomaterial‐inspired architectures are divided into six categories, and the design–mechanic–function relationships of the structures are described. In addition, the preparation methods and applications of these architectures are briefly presented.

In this review, our main purpose is to discuss the recent theoretical and experimental progress in the field of material strengthening by investigating the microstructure architecture in advanced materials. We explored in detail the strengthening mechanism of six major types of microstructure architectures, and briefly introduced the relevant preparation methods and application prospects (**Figure** **2**). Specifically, we discuss and compare the functionality, applicability, and coupling of the microstructure architecture, providing a reference for the optimization and selection of the architecture. Finally, we bring forward‐looking views on the work that may need to be developed in this field in the future, as well as highlighting their important significance for material development and engineering application.

## 2 Homogenous and Inhomogeneous Architecture

### 2.1 Homogeneous Architecture

Homogeneous architecture is a kind of dense and uniform microstructure formed by various phases, reinforcing particles, and grains of materials.^[^ ^112^, ^113^, ^114^, ^115^ ^]^ The architecture achieves a breakthrough in material performance by combining the excellent performance of various components with the refinement and uniformity of the microstructure.^[^ ^116^, ^117^, ^118^, ^119^ ^]^ The problems to be solved include the inhibition of rapid grain growth, refinement of structure, uniform distribution of components, and good interfacial bonding.^[^ ^120^, ^121^, ^122^, ^123^, ^124^, ^125^ ^]^ Besides, the mechanical and physical properties of homogeneous structures are the result of the combined action of various strengthening mechanisms. The dense and uniform microstructure can effectively improve the strength, toughness, electrical conductivity, and thermal conductivity of materials.

In the machining processes of homogeneous architecture, reinforcement is prone to deformation, aggregation, and folding, which brings a series of dispersion problems to reinforcement. Therefore, the preparation of homogeneous architecture requires the combination of external force, surface chemical treatment, mixed components, and preparation conditions to achieve uniform distribution of reinforcements.^[^ ^126^, ^127^, ^128^, ^129^, ^130^, ^131^ ^]^ Typical preparation methods include stirring casting, powder processing, in situ synthesis, and molecular‐level mixing. Stir casting is a method of producing homogeneous materials in the liquid phase in which the reinforcement is uniformly dispersed in the matrix by mechanical agitation.^[^ ^132^, ^133^ ^]^ The process is mainly aimed at composites with large‐sized reinforcements, and the process is relatively simple and economical. However, there are many disadvantages of this process, such as the wettability between the two materials and the possible chemical reaction, as well as the easy occurrence of voids in the as‐cast composite materials. Powder processing generally refers to the production of uniformly mixed micron‐ or nano‐sized powders from raw materials through a powder‐making process, and then the use of densification technology to produce bulk materials.^[^ ^134^, ^135^ ^]^ Generally, to improve the dispersion state of powder, mechanical ball milling, suspension magnetic stirring and supergeneration are used to uniformly disperse the mixed powder. The disadvantage is that the mechanical force may damage the structure of the reinforcing material and introduce impurities. The densification technologies include compact sintering, spark plasma sintering, cold isostatic molding, and hot isostatic molding. The basic properties of the mixed powder (fluidity, compressibility, particle size, and composition, etc.) and the densification conditions (temperature and pressure, etc.) can directly affect the quality of the compacts.

To overcome the damage and pollution of the material structure caused by mechanical force and chemical dispersion methods, scientists used in situ synthesis technology to pretreat the material. For metal matrix composites, this technology is generally to directly synthesize the uniformly distributed fine reinforcement phase on the metal matrix by adding appropriate reactants (gas phase, liquid phase, and solid phase powders).^[^ ^136^, ^137^ ^]^ For polymer matrix composites, this technology is generally used to graft polymer molecules onto the surface of reinforcing materials by adding appropriate initiators.^[^ ^138^, ^139^ ^]^ This preparation technology ensures the good combination and uniform dispersion of reinforcement and matrix, but the relatively low production efficiency limits its application in mass production. In addition, the separation and processing in the post‐treatment process make the preparation technology lack stability and controllability. Molecular‐level mixing refers to the attachment of small nanoparticle materials to functionalized carbon nanomaterials.^[^ ^140^, ^141^ ^]^ This method is conducive to interfacial bonding and uniform dispersion of materials. However, due to the introduction of a large number of defects on the surface of functional groups of carbon nanomaterials, the mechanical and physical properties of the composites have been greatly affected. Moreover, the control of the oxidation and reduction steps is a challenge as well.

**Figure 3:**
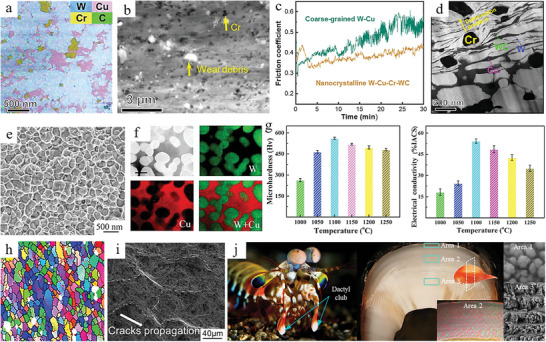
Homogeneous architectures with uniform distribution of reinforcement in the matrix. a–d) Element distribution, surface wear morphology, friction coefficient and deformation gradient of nanocrystalline W–Cu composites. Reproduced with permission.^[^ ^149^ ^]^ Copyright 2021, Elsevier B.V. e–g) Scanning electron microscope (SEM) image, elemental distribution, microhardness and electrical conductivity of W–Cu compacts. Reproduced with permission.^[^ ^150^ ^]^ Copyright 2020, Elsevier B.V. i) EBSD map of grain structure at 300 °C. Reproduced with permission.^[^ ^151^ ^]^ Copyright 2021, Elsevier Ltd. j) The transverse section of the dactyl club consists of the impact surface (area 1), impact region (area 2) and periodic region (area 3). Reproduced with permission.^[^ ^90^ ^]^ Copyright 2020, Springer Nature.

After many years of development, there are still many problems to be improved in the composite materials with uniform microstructure architecture. It has been shown to be effective in introducing alloying elements to refine the microstructure of composites.^[^ ^142^, ^143^, ^144^ ^]^ This composite material combines the excellent properties of various components, and the introduced metal elements can refine the grain structure by solution segregation and phase separation effect.^[^ ^145^, ^146^, ^147^, ^148^ ^]^ However, it is still a challenge for the doped metal elements to be uniformly distributed in the matrix and form ultra‐fine structures. Taking the W–Cu matrix composite as an example, the homogeneous and compact W–Cu alloy has excellent electrical, thermal and mechanical properties. Li et al. prepared a W–Cu‐based nanocomposite co‐doped with Cr and WC.^[^ ^149^ ^]^ From the results of super‐EDS analysis, it can be seen that the microstructure has a uniform W and Cu phase distribution, which is attributed to the nanostructure of the in‐situ synthesized nanocomposite powder being maintained after sintering (**Figure** **3**a). Moreover, the uniform distribution of Cr and WC nanoparticles in the W–Cu matrix can significantly improve the hardness of the composite. Compared to coarse‐grained W–Cu composites, nanocrystalline W–Cu composites with higher hardness have lower and more stable coefficients of friction. Not only that, the uniform distribution of refined Cr and WC phases makes the stress distribution on the wear surface uniform and suppresses the initiation of local cracks (Figure 3b,c). In the plastic deformation zone after wear, a strain gradient is formed between the hard phase (Cr and WC) and the soft phase (W and Cu), and a large number of geometrically necessary dislocations are generated, making the wear surface hardened (Figure 3d).

Although the above W–Cu composites refine the reinforcing phase, there are obvious differences in the shape and size of the W, Cu, Cr, and WC phases. These differences affect the mechanical properties and conductivity of the composites to some extent. To overcome the irregular shape and difficulty to control the grain growth of W–Cu powder in W–Cu composites, a strategy of core–shell W–Cu nanoparticles was developed by Li et al.^[^ ^150^ ^]^ At 1100 °C, polyhedral W grains embedded in a continuous Cu network formed a homogeneous microstructure, and this uniform dense structure was also evident from the distribution of elements (Figure 3e,f). The formation of the microscopic homogeneous structure is attributed to the fact that liquid Cu fills the voids and forms a network around the W grains when the sintering temperature is higher than the melting point of Cu, effectively suppressing the coarsening of the W grains. Therefore, the microhardness and electrical conductivity of the composites obtained at 1100 °C are the highest due to the high densities and small grain sizes of the composites at this temperature (Figure 3g). The homogeneous Cu network inside the composites is the main reason for the high conductivity obtained.

The uniform microstructure was prepared by the in situ synthesis method mentioned above, and the compatibility of the matrix phase and reinforcement phase was good. In addition, severe plastic deformation is also a common method to uniformly distribute and refine particles. Therefore, the combination of in situ synthesis and mechanical force is a good strategy to prepare composites with uniform distribution and fine particles. Zhao et al. used elliptical cross‐section torsion extrusion (E‐TE) to prepare TiB_2_ particle‐reinforced Al‐Zn‐Mg‐Cu‐based composites.^[^ ^151^ ^]^ The composites treated by the E‐TE process had a homogeneous architecture and the finest grain structure at 300 °C (Figure 3h,i). The purpose of refining and homogenizing the microstructure of the composites is to obtain good properties with both strength and ductility. By performing uniaxial tensile tests on E‐TE‐treated samples, the ultimate strength and elongation of the samples are improved.^[^ ^111^ ^]^ Besides, small equiaxial dimples and obvious ridges after step crack extension can be found on the fracture surface of the samples, which indicates that the samples are ductile fractures and TiB_2_ particles are uniformly distributed (Figure 3i). The homogeneous microstructure architecture hinders crack propagation and creates different crack propagation paths, which in turn improves the plasticity of the samples.

Homogeneous architectures have obvious advantages in increasing the strength‐toughness of materials, so some animals in nature also adopt this architecture. In recent years, scientists have found that the impact surface of mantis shrimp's dactyl club is a homogeneous architecture composed of hydroxyapatite nanoparticles and an organic matrix.^[^ ^90^ ^]^ The impact surface is about 70 µm thick, with a dense pack of hydroxyapatite nanoparticles of about sub‐100 nm and a packing density of about 88 vol% (Figure 3j). Microimpact experiments were performed on the impact surface using spherical and cubic angle indenters. Particle packing was observed in the impact surface, and particles were worn off the impact surface after 100 impacts. Furthermore, the penetration depth in the presence of the impact surface was reduced by half compared to that without the impact surface at different loads. This indicates that the microstructure architecture of the impact surface is able to locate the damaged area and prevent crack initiating and extension. Further studies showed that high strain rate or impact induced dislocation formation, amorphization, particle changes (breakage, rotation, and translation) and organic phase changes (plastic deformation, fibril bridging) are effective energy absorption mechanisms. To verify this energy dissipation mechanism, the strain rate of the bicontinuous network was simulated at the nanoscale and the energy dissipation of grain boundary fracture was simulated at the atomic scale. Hydroxyapatite nanoparticles deform more uniformly at high strain rates, localize deformation at low strain rates, and increase stiffness and strength with increasing strain rates. Moreover, small‐angle grain boundaries lead to a decrease in the overall strength of hydroxyapatite nanoparticle crystals. This homogeneous architecture composed of a hard mineral phase and a soft organic phase brings dawn to the preparation of impact‐resistant materials with high strength and toughness.

The preparation methods for homogeneous architectures are more abundant, such as casting, additive manufacturing, powder metallurgy, and in situ synthesis.^[^ ^152^, ^153^, ^154^, ^155^, ^156^, ^157^, ^158^ ^]^ However, the key to improving the performance lies in the density and refinement of the reinforcement, the uniformity of distribution, and the bonding strength of the interface. In addition, the integrity of the structure, shape, and composition of the reinforcing material should be guaranteed during the processing, which will greatly affect the mechanical properties and other functions of the composite. For particle‐reinforced composites, the refinement of the reinforcing particles leads to agglomeration of the particles in the matrix.^[^ ^159^, ^160^ ^]^ Moreover, the density of the reinforcing particles also affects the strength and toughness of the composite. Therefore, reasonable regulation of the density, degree of refinement, and homogeneity of the reinforcing phase is a factor to be considered for the design of homogeneous architectures. Furthermore, some preparation methods inherently have certain defects, such as the casting process may lead to voids inside the composite and powder metallurgy may destroy the structural integrity of the reinforcement. Therefore, it is also important to enhance the preparation techniques on the basis of the reasonable design of the reinforcement phase.

In a nutshell, the improvement of mechanical properties of homogeneous architectures mainly depends on the inherent physicochemical properties of reinforcement and matrix as well as the size, density, and dispersion state of reinforcement. The uniformly dispersed reinforcement can make the stress uniformly distributed, prevent crack initiation and propagation, and improve the strength and toughness of the composite. When the size of reinforcement is small, the stress distribution is more uniform, and it is difficult to form high local stress. In addition, the reinforcement blocks the crack propagation path and makes the crack diverge and deflect. For metal matrix composites, the uniformly distributed fine grains mainly strengthen the composites by increasing the barrier of grain boundaries to dislocations. The increase of dislocations at grain boundaries significantly improves the strain‐hardening ability of the material. Furthermore, the more uniform and smaller the precipitated phase distribution of the metal matrix composite, the more obvious the strengthening effect of the composite. The precipitated phase hinders the dislocation slip and improves the deformation resistance of the composite. However, for different types of materials, the strengthening mechanism of homogeneous architecture is different. Here, homogeneous architecture is mainly applicable to composites that need to improve mechanical properties. Additionally, the homogeneous architecture can also be used for composites that need to improve conductivity, thermal conductivity, and wear resistance.

### 2.2 Inhomogeneous Architecture

In addition to the excellent performance of homogeneous architectures in improving the properties of composites, well‐designed inhomogeneous architectures can also play an unexpected role. The inhomogeneous architecture described in this section refers to the distribution of various phases and components of the composite in a non‐uniform form in the matrix, which distinguishes it from the inhomogeneous architecture with a typical structure introduced in the later sections. The strengthening of the inhomogeneous architecture depends mainly on the basic properties, characteristic dimensions, volume fractions, and distribution characteristics of the various components and phases.^[^ ^161^, ^162^, ^163^, ^164^ ^]^ The main objective is to overcome the paradox of strength and ductility in composites by suppressing the initiation and propagation of local cracks.

**Figure 4:**
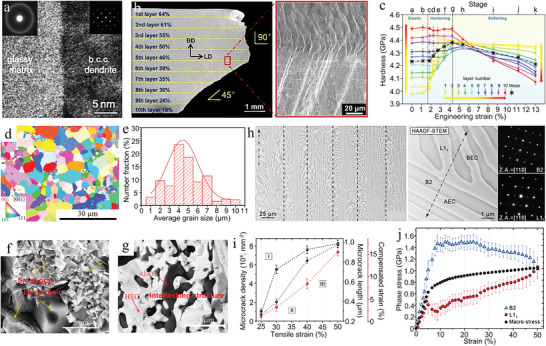
Inhomogeneous architectures with nonuniform distribution of reinforcement in the matrix. a,b) High‐resolution transmission electron microscopy (HRTEM) micrograph of the interface between the two phases. c) Fracture morphology of gradient BMGC 1‐10 layers and microhardness evolution of A‐K segment corresponding to layers 1‐10. a–c) Reproduced with permission.^[^ ^165^ ^]^ Copyright 2021, Elsevier Ltd. d,e) Microstructure and grain size distribution of Al_2_O_3_/(NbTaMoW)C composites. f,g) Fracture morphology of the composites and the distribution of Al_2_O_3_ in the matrix. d–g) Reproduced with permission.^[^ ^166^ ^]^ Copyright 2021, Elsevier Ltd. h) Eutectic high‐entropy alloy (EHEA) with herringbone microstructure contains B2 and L1_2_ phases. i) The relationships among microcrack density, microcrack length, and tensile strain in the AEC. j) Stress partitioning of B2 and L1_2_ phases in situ synchrotron experiments. h–j) Reproduced with permission.^[^ ^170^ ^]^ Copyright 2021, American Association for the Advancement of Science.

Functional gradient structures belong to the more classical inhomogeneous architectures, which can synergistically strengthen composites based on the interactions between adjacent layers and the asynchronous deformation of the non‐uniform structure. For example, Lu et al. fabricated multilayered in situ bulk metallic glass composites (BMGCs) using laser additive manufacturing techniques.^[^ ^165^ ^]^ The purpose of this design is to mitigate the elastic mismatch between adjacent layers by varying the strength layer by layer. Changing the strength is achieved by adjusting the volume fraction of the crystal dendrites. The mixture of glass matrix and crystalline dendrites can be clearly seen near the interface region of glass dendrite (**Figure** **4**a). Uniaxial tensile experiments were performed on BMGC with a fracture angle of 90° from layer 1 to layer 5 and 45° from layer 6 to layer 10. From layer 1 to layer 10, the microhardness gradually increased as the dendrites decreased. When the BMGC is subjected to tensile forces, there is a strain distribution between the soft and hard layers, resulting in a completely opposite microhardness gradient after deformation (Figure 4b,c). By the above analysis, the strengthening effect of this gradient architecture is applicable to many other composites, and the synergistic deformation of soft and hard phases can effectively improve the overall toughness. Moreover, the interlocking structure formed by various phases with non‐uniform distribution can be used as an inhomogeneous architecture to enhance the mechanical properties of composites. The interlocked structure is different from the gradient structure mentioned above. The interlocked structure makes the fracture toughness of the composite basically consistent in all directions. This high fracture toughness is mainly due to the crack deflection, bridging, and pull‐out of the composite in all directions caused by the inhomogeneous distribution and interlocking reinforcement phases. Liu et al. prepared Al_2_O_3_/(NbTaMoW)C composites by in situ exothermic reactions, whose microstructure consists of Al_2_O_3_ phase and (NbTaMoW)C phase with adjacent grains having different crystal orientations (size range of 1–10 µm) (Figure 4d,e).^[^ ^166^ ^]^ Small grains are observed randomly distributed between the large grains on the fracture surface. This inhomogeneous distribution of grain sizes allows the crack to deflect when it encounters the grains. Furthermore, the liquid phase of Al_2_O_3_ wrapped around the high entropy carbide (HEC) at 1600°C to form an interlocking structure (Figure 4f,g). Therefore, the synergistic effect of crack deflection and interlocking structure improves the flexural strength and fracture toughness of the composites.

The cracks of composites are usually caused by localized plastic deformation, and unstoppable crack propagation can cause premature failure of the material.^[^ ^167^, ^168^ ^]^ Inspired by biological structures, scientists have been able to design inhomogeneous architectures that can effectively buffer and prevent crack propagation.^[^ ^169^, ^170^, ^171^ ^]^ Here, the strengthening mechanism of bionic inhomogeneous architecture composites is illustrated by the example of herringbone eutectic high‐entropy alloy.^[^ ^170^ ^]^ The Al_19_Fe_20_Co_20_Ni_41_ eutectic high‐entropy alloy prepared by directional solidification consists of aligned eutectic colonies (AEC) and branched eutectic colony (BEC) lamellae (Figure 4h). The herringbone microstructure contains the L1_2_ phase (soft, face‐centered cubic) and the B2 phase (hard, body‐centered cubic). The microcrack density increases rapidly at 25–30% strain, followed by a slow increase (30–50%). Moreover, the microcrack density and length increase very slowly at 40–50% strain (Figure 4i). In the AEC and BEC regions, when the microcracks penetrate the B2 hard phase, they are passivated in the L1_2_ soft. This implies that the alternating soft‐hard phases are able to withstand the high‐density cracks and effectively passivate the crack tips and buffer the crack propagation. According to the synchrotron high‐energy X‐ray diffraction (SHE‐XRD) results, the yield of the L1_2_ soft phase makes the stress distribution of the B2 hard phase increase rapidly. At a strain of 25%, the B2 hard phase undergoes a slow decrease in stress, and the strain hardening of the L1_2_ soft phase makes the undergoing stress increase slowly (Figure 4j). Therefore, the design of this herringbone inhomogeneous architecture enables the composite to have excellent fracture toughness.

The above analysis shows that the design mentality of these special inhomogeneous architectures is basically similar to the classical inhomogeneous architectures mentioned below. Prevention of crack initiation and propagation is the primary objective of microstructure design and distribution. In short, the excellent performance of the inhomogeneous architecture is due to the special distribution and properties of the reinforcement and matrix. Different types of fracture occur in the hard phase and soft phase of the composite, and the incompatible deformation caused by this composite fracture will increase the stress in the reinforcement. Non‐uniform distribution of reinforcement will lead to multipath, multitime, multistage, and multiregion characteristics of material failure. In addition, the inhomogeneous distribution of reinforcements blocks the movement of dislocations and improves the strain‐hardening ability of the matrix. Furthermore, the coordination of the size, distribution, and content of soft and hard phases is also the key to determining the final properties of the composites. Therefore, this kind of inhomogeneous architecture can greatly improve the overall performance or single‐aspect performance after careful design. Here, several inhomogeneous architectures introduced in this review are mainly applicable to composites that need to improve mechanical properties, but the applications don't stop there. Additionally, the application range of inhomogeneous architecture is very wide, which can be applied to composite materials in aerospace, automotive, microelectronics, and other fields. Inhomogeneous architectures have incomparable advantages over homogeneous architectures in some aspects. The designability of inhomogeneous architectures enables them to meet some special requirements, such as functional gradient, multiple length scales, and thermal barrier coating materials.

## 3 Lamellar Architecture

**Figure 5:**
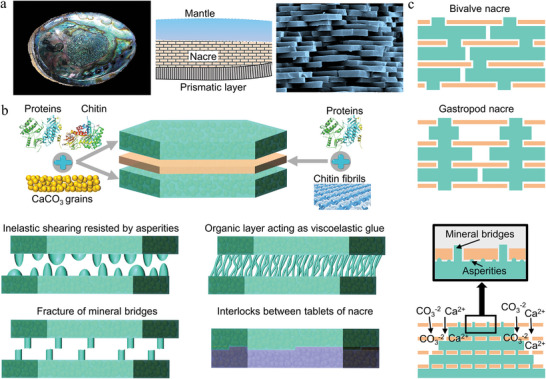
The composition, growth, and strength‐toughness mechanisms of the nacre layer. a) The shell of bivalve mollusk is composed of periostracum, prismatic layer, and nacre. An enlarged view of the brick and mortar structure in the nacre is also shown. Reproduced with permission.^[^ ^182^ ^]^ Copyright 2008, Elsevier Inc. b) The structural composition of nacre and the types of strengthening and toughening between bricks. c) Types of nacre and the growth mechanism.

In the bionic laminar architecture, the nacre has been heavily studied for its ability to combine strength and toughness to allow it to withstand large inelastic deformations. The nacre is found in the shells of bivalves and gastropod mollusks and consists of an aragonite layer and an organic layer.^[^ ^172^, ^173^, ^174^, ^175^ ^]^ Specifically, the aragonite layer consists of CaCO_3_ particles, proteins, and chitin, and the organic layer consists of proteins and chitin fibrils. The good toughness and impact resistance of the nacre mainly depend on the viscoelastic organic layer to provide a path for crack deflection.^[^ ^176^, ^177^, ^178^ ^]^ Besides, the formation of mineral asperities and mineral bridges between the aragonite layers, the deformability of the organic layers, and the structural interlocking of the aragonite layers are all enhanced toughening mechanisms to resist shear deformation (**Figure** **5**a,b).^[^ ^9^, ^173^, ^179^, ^180^, ^181^, ^182^ ^]^ Therefore, composite materials with nacre‐like structures can achieve both strength and toughness. However, we often neglect the growth mechanism of the nacre structure. The biological knowledge of nacre formation is of great significance for the preparation of biomimetic composites. Mollusks periodically form organic layers, and the chitin network provides the basic requirements for mechanical strength. The organic layer membrane allows the passage of CO_3_^−2^ and Ca^2+^ and the formation of aragonite layers between the organic layer membranes.^[^ ^173^ ^]^ The aragonite layers grow laterally between each other and meet each other, at which point growth stops and a thin organic layer forms at the meeting point (Figure 5c).

Inspired by the nacre structure, composites with carbon nanotubes and graphene as reinforcement layers are more typical of the laminar architecture of materials.^[^ ^183^, ^184^, ^185^, ^186^ ^]^ Carbon nanotubes are stacked in a random distribution or specific orientation to form 2D reinforcement layers. The laminar architecture composites composed of reinforcement and matrix layers have excellent mechanical properties. Yang et al. investigated the fracture behavior of graphene/Ni composites under uniaxial tensile loading by means of molecular dynamics simulations.^[^ ^187^ ^]^ At a strain of 5.4%, the main crack extended into the first graphene layer and caused an abrupt drop in stress. Thereafter, the crack width keeps getting larger with increasing strain and the stress concentration at the crack tip is released. At strains of 22.7–90.4%, as the crack expands, the graphene layer blocks the natural expansion of the crack leading to crack penetration through the metal layer from the weak point of the graphene layer. Moreover, we found that the increase in load leads to dislocations nucleating at the metal layer and the interface. The stress release at the crack tip is attributed to the sliding of metal layer atoms on the graphene surface. Therefore, graphene/Ni composites can be toughened by preventing crack propagation. Besides, we need to further understand the influence of the structure and distribution characteristics of graphene on the properties of graphene/nickel composites on the basis of the above. The research shows that the structural alignment and stress transfer between the graphene layers and the metal matrix is also an important aspect of enhanced toughening. The minimum distance between graphene layers that can increase the stiffness and modulus of graphene nanocomposites was shown to be 3.05 nm by Santhapuram et al.^[^ ^188^ ^]^ Furthermore, when the distance between graphene layers was 10, 15, and 20 nm thick, the composites exhibited greater flexibility. The increase in the bending modulus of the composites was related to the graphene quality. By comparing the effect of pristine graphene and polycrystalline graphene on the bending modulus of the composites, we found that the higher interfacial shear strength of polycrystalline graphene in the matrix resulted in a 15–20% increase in the bending modulus. Furthermore, the stress distribution in the polycrystalline graphene nanocomposites exhibited higher tensile stress and lower compressive stress, which may be related to the interfacial shear strength.

In addition to graphene as a hard phase in laminar architecture composites, the research on carbon nanotubes (CNT) as a layered structure in metal matrix composites has also received attention. Meng et al. reported microscale CNT/Cu laminar composites prepared by electrophoretic deposition of carbon nanotubes on metal surfaces to prepare laminar units, followed by hot pressing and hot rolling.^[^ ^189^ ^]^ The strength of the composites was increased to 183 MPa and the ductility was increased to 30.9%. The key to obtaining such excellent mechanical properties is the uniform dispersion of carbon nanotubes and strong interfacial bonding. Both the inherent high strength of the carbon nanotube lamellar structure and the dislocation‐blocking effect induced at the interface can effectively improve the strength of the composite. Microscopically, CNTs are distributed at the grain boundary, which improves the dislocation storage capacity at the grain boundary. Under the combined action of geometrically necessary dislocation and back stress, the strength and toughness of the composite are strengthened. Moreover, the carbon nanotube layers facilitate the load transfer and hinder the strain localization. Similar to the deformation of the nacre, cracks bypass the carbon nanotubes to deflect in the softer Cu matrix. Therefore, energy dissipation also exists in laminated CNT/Cu composites.

In addition to being widely used in the above‐mentioned metal matrix composites, the lamellar architecture is also used to improve the fracture toughness of ceramic matrix composites. Ceramic matrix composites are widely used because of their high‐temperature resistance and high strength.^[^ ^190^, ^191^ ^]^ However, the poor fracture toughness of such composites hinders their wide application. A ZrB_2_‐SiCw/Ti‐Nb‐Ti laminated composite was proposed by Bai et al.^[^ ^192^ ^]^ The structural unit of the laminated ZrB_2_‐SiCw/Ti‐Nb‐Ti composite consists of alternately stacked ZrB_2_‐SiCw matrix ceramic layer and Ti‐Nb‐Ti metal interlayer. The cracks in the ZrB_2_‐SiCw/Ti‐Nb‐Ti laminated composites exhibit a jagged shape compared to the ZrB_2_‐SiCw ceramics. In the (Ti, Nb) reaction layer, crack propagation in the ceramic layer was not hindered, and the changes about crack propagation direction and crack bridging ware observed. In conclusion, the improved fracture toughness of ZrB_2_‐SiCw/Ti‐Nb‐Ti laminated composite is mainly due to the fact that both crack propagation behavior (deflection, bridging and bifurcation) and plastic deformation (slip, dimples, and tear ridges) can dissipate a large amount of energy. From the results of the indentation load resistance test of the samples, the slope of ZrB_2_‐SiCw is much steeper than that of ZrB_2_‐SiCw/Ti‐Nb‐Ti. Therefore, the laminated samples were able to maintain the strength well as the indentation load increased. Besides, the curves of toughness of the ZrB_2_‐SiCw/Ti‐Nb‐Ti composites showed good damage tolerance due to the effect of several toughening mechanisms. From the load–displacement curves and fracture morphology of the samples, the fracture of the ZrB_2_‐SiCw/Ti‐Nb‐Ti composites occurred layer by layer, resulting in a step‐like load–displacement curve and a fracture morphology with uneven fluctuating fractures. Therefore, this layer‐by‐layer fracture effect is beneficial to dissipate more fracture work and improve the toughness.

With the progress of preparation technology, the layered architecture is no longer limited to the simple layer‐by‐layer stacking. The design of layered structure began to focus on the microstructure of each layer and the twist angle between layers, which provided hope for further improving the performance of layered composites. Currently, the rapid development of 3D printing technology makes it a preferred choice for manufacturing composites. Li et al. prepared the discontinuous interpenetrating phase composites (d‐IPC) with the digital light processing (DLP) 3D printer.^[^ ^193^ ^]^ The principle of preparation revealed that the discontinuous pillar layers were formed using yttrium oxide particles printed layer by layer, and the layers can be rotated by 90°. Quasi‐static compressive stress‐strain studies were performed on pure polymer, 0.5, 1, and 2 wt% d‐IPC samples, and the results showed that the strength of 1 wt% d‐IPC samples was higher and appeared gradual plateau regions. Therefore, the 1 wt% d‐IPC samples are suitable to be prepared as ideal energy‐absorbing materials.

**Figure 6:**
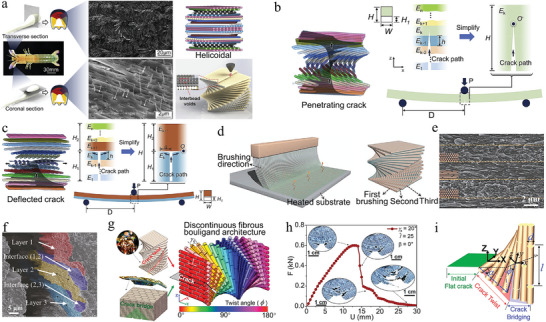
Design, preparation, and strength‐toughness mechanisms of the Bouligand architecture. a) SEM images of the periodic region in mantis shrimp's dactyl club and models of bioinspired composite laminate. b,c) Simplified mechanism of the propagation of penetrating crack and deflection crack. a–c) Reproduced with permission.^[^ ^194^ ^]^ Copyright 2020, Elsevier Ltd. d,e) Xonotlite nanowires (20 wt%)–sodium alginate composite film prepared by continuous brushing. f) Crack propagation path in the twisted plywood materials. d–f) Reproduced with permission.^[^ ^195^ ^]^ Copyright 2018, Oxford University Press. g) Printing discontinuous fibrous Bouligand architecture based on the combined features of Bouligand and nacre. h) Force–displacement curve and crack propagation path of the sample. i) Fracture mechanics model of crack twisting and bridging in discontinuous fibrous Bouligand structure. g–i) Reproduced with permission.^[^ ^196^ ^]^ Copyright 2022, National Academy of Science.

Furthermore, the Bouligand structure is a biological structure similar to the laminar architecture of d‐IPC composites, which is composed of laminated chitin fibers in a protein matrix. The Bouligand structure has unusual toughness and resistance to damage, for which scientists have made considerable efforts to understand the mechanisms involved. Yin et al. used the Bouligand structure of the periodic region of the mantis shrimp as a target, and investigated the strengthening mechanism of the bio‐inspired laminated composite (**Figure** **6**a).^[^ ^194^ ^]^ The fracture resistance of laminate composites mainly lies in the deflection or penetration of cracks in the layered region. Thus, based on the microstructure and failure mode of the composite, a uniform 2D beam model was developed for the analysis of two cases of crack propagation (Figure 6b,c). The conditions for determining whether cracks will penetrate or deflect into the interface are

(1) $$\left\{\begin{array}{cc} \frac{G_{P}}{G_{D}}>\frac{G_{\mathrm{PC}}}{G_{\mathrm{DC}}} & \mathrm{Penetrating}\;\mathrm{crack}\\ \frac{G_{P}}{G_{D}}<\frac{G_{\mathrm{PC}}}{G_{\mathrm{DC}}} & \mathrm{Deflected}\;\mathrm{crack} \end{array}\right.$$

where *G*_PC_ and *G*_DC_ are the fracture toughness values of the lamella and interface. *G*_P_ and *G*_D_ are the energy release rates of the crack penetrating and deflected at the lamellae.

A theoretical model based on the plane strain assumption can be used to identify whether cracks in Bouligand laminated structures will penetrate or deflect. The theoretical model correlates the interlaminar angle with the potential for cracking and is used to select the best lamination solution to resist fracture of the Bouligand laminate structure. Inspired by this Bouligand structure with excellent fracture resistance, Chen et al. constructed micro/nanofibers into inorganic–organic 2D films and laminated them into macroscopic 3D bulk composites.^[^ ^195^ ^]^ The programming and continuous preparation of HA‐SA composites in sodium alginate (SA) matrix were achieved due to the alignment of hydroxyapatite (HA) mineral ultrafine fibers in the same direction as each brushing (Figure 6d,e). HA‐SA composites have excellent fracture toughness since cracks always follow the twisted path between the HA‐SA layers. The stresses at the crack tips preferentially bypass the fibers rather than cut off, so that the main crack shows a complex twist and many microcracks at the tips (Figure 6f). This small‐angle twisted plywood structural material successfully replicates the natural laminar architecture and toughening mechanism.

The characteristics and strengthening mechanisms of the lamellar architecture of mantis shrimp and abalone are presented above. The battle for survival between mantis shrimp and abalone has resulted in the possession of exquisite defense structures for each other. It may be a good strategy to combine the Bouligand laminated structure of mantis shrimp with the brick and mortar structure of abalone. Inspired by this, Wu et al. designed a discontinuous fiber Bouligand (DFB) architecture by combining the two structures of Bouligand and nacre (Figure 6g).^[^ ^196^ ^]^ The DFB composite exhibited total energy dissipation insensitivity to the initial crack direction *β* in a three‐point bending test. At different pitch angles *γ*_0_, the crack direction insensitivity remains constant in the DFB structure. The force–displacement curves clearly show the crack propagation paths including crack twisting and fiber bridging in the DFB composites (Figure 6h). Figure 6i elucidates the fracture mechanics model of the DFB composite for explaining the mechanism of crack direction insensitivity and maximum energy dissipation in the structure. In conclusion, this DFB structure with the combination of Bouligand and nacre structure has significantly enhanced fracture resistance and crack direction insensitivity. In addition, the proposed strategy to optimize the structure design based on pitch angle, discontinuous fiber length, crack bridging fracture energy, and twist angle distribution is scalable.

To summarize, the strengthening mechanism of laminar architecture is mainly to prevent crack propagation in the form of crack deflection. The deflection of the crack promotes the release of energy at the crack tip and leads to the increase and bending of the crack propagation paths. And then, the composite material shows the characteristics of multiple cracks and progressive fracture, which improves its damage tolerance and toughness. Furthermore, the strain redistribution, crack bridging and structural interlocking between the multilayer structures consisting of soft and hard phases are the reasons for the high strength and toughness of the composites. The redistribution of strain can optimize the transfer of load and prevent crack initiation and propagation. The bridging of crack can be used as an effective energy dissipation way to improve the toughness of composites. The interlock of structure can prevent the debonding of reinforcement and improve the strength of composite materials. In addition to those mentioned above, the design of multiscale, multishape and gradient for laminar architecture is a strengthening and toughening mechanism that needs further investigation. The multiscale and multishape designs of lamellar platelets can enhance the structure interlocking and stress distribution, and provide more paths for crack deflection. Further, the gradient design of the lamellar platelet can be mainly based on the thickness and density, which is mainly used to adjust the strength and toughness of the composite. Here, the lamellar architecture is mainly applicable to composite materials that need to improve strength, toughness, fatigue resistance, and impact resistance, but it is not limited to this.

## 4 Columnar Architecture

**Figure 7:**
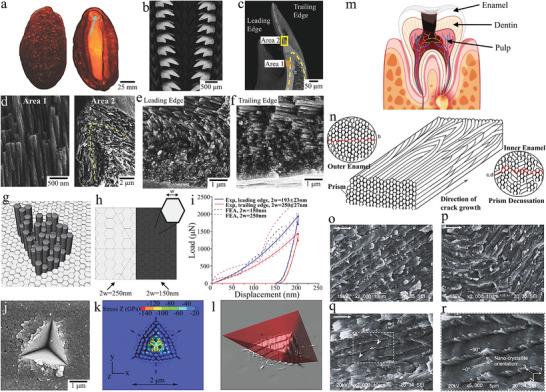
The microstructure, strength, and toughness of the columnar architecture of chiton and human teeth. a,b) Chiton and their teeth arrangement. c–f) The highly mineralized shell and organic‐rich core of Chiton teeth. In addition, the alignment direction and continuity of the nanorods in the leading edge of the shell (area 1) and the nanorods at the apex of the longitudinal fracture (area 2) are also shown. The longitudinal fracture surfaces of the leading and trailing edges are transformed from dense nanoparticle layers to nanorods oriented parallel to the tooth surface. g,h) The tooth microstructure was idealized as the hexagonal rod, and the finite element models of the trailing (250 nm) and leading (150 nm) edges were established. i–l) The comparison of the indentation experiment and finite element simulation on the leading and trailing edges highlights the stress contour, material removal, and crack propagation. a–l) Reproduced with permission.^[^ ^201^ ^]^ Copyright 2014, Wiley‐VCH. m,n) Composition of teeth and microstructure of outer enamel and inner enamel. o–r) Crack propagation, crack deflection, crack distortion, fracture, and nanocrystallites orientation in prisms of enamel. m–r) Reproduced with permission.^[^ ^202^ ^]^ Copyright, 2009 Elsevier Ltd.

Chiton, human teeth, and nacre have a layer of columnar architecture composites, which naturally evolved to be highly hard, ductile, and wear‐resistant depending on their survival needs.^[^ ^197^, ^198^, ^199^, ^200^ ^]^ These columnar architectures of composites use the hard prism and the soft matrix to resist the fatigue load and deformation. Grunenfelder et al. investigated the structure‐property relationships of chiton teeth (**Figure** **7**a,b).^[^ ^201^ ^]^ The longitudinal fracture surfaces of the teeth were carefully observed to fully understand the mechanism of the action of tooth microstructure on strength and toughness (Figure 7c–f). For the fracture surface of the leading edge of the tooth (area 1), closely aligned hexagonal nanorods parallel to the tooth can be clearly observed. Furthermore, the nanorod region is transformed from the dense nanoparticle region in the outermost layer of the tooth, and the rotation phenomenon of the nanorod occurs. At the apex of the tooth tip (area 2), the nanorods are parallel to the tooth contour and wrap around the entire shell. For the fracture surface of the trailing edge of the tooth, the nanorod region is transformed from the thinner nanoparticle region and there is no rotation of the nanorod. To further understand the micromechanics of the nanorod structure, the elastic‐plastic properties of single‐crystal magnetite were used to model the nanorod (Figure 7g,h). The simulation results of the nanoindentation experiment show that the total volume of energy dissipated at the interface is larger for small rods than for large rods at a penetration force of about 160 nm (Figure 7i). Moreover, cracks were found in the transverse polished portion and propagated around and between the mineralized nanorods in both experimental and simulated results of high‐load nanoindentation. Small radial cracks were observed in the edge region and material removal occurred around the plane of the sharp indenter (Figure 7j–l). Thus, we find that the tooth architecture of the chiton evolved in response to their feeding needs, and that it has both high hardness and toughness to allow it to resist intermittent impact and abrasion.

Human teeth also have similar structures, but they are some differences. The human tooth consists of the enamel, dentin, and pulp in order from the outside to the inside, with the enamel consisting of prisms formed of hydroxyapatite and organic matter formed of proteins (Figure 7m).^[^ ^202^ ^]^ These prisms extend in a parallel manner from the dentin‐enamel junction to the outer surface of the enamel. To meet the daily dietary needs of humans, enamel must be sufficiently hard. To assess the effect of the columnar architecture of enamel on fracture toughness, Bajaj et al. conducted in‐depth research on the resistance behavior of crack propagation for enamel. The prisms of the outer enamel were neatly aligned in parallel, while the prisms of the inner enamel showed a crossover (Figure 7n). In the outer enamel, cracks propagated along the weaker prism boundaries simply because of the presence of soft organic matter at the boundaries (Figure 7o). In the inner enamel, the crack path is deflected with the inclination of the prism (Figure 7p). In this region of acute crossover, the deflection, twisting, and fracture of the prism impede crack propagation. Moreover, the fracture of the prisms leads to the pull‐out and fracture of the nanocrystals (Figure 7q,r). Thus, the toughening mechanism of the columnar architecture of enamel is mainly reflected in the inner enamel region, where parallel and crossed prisms are the main toughening structures that promote crack bridging and deflection as well as inhibit crack propagation.

**Figure 8:**
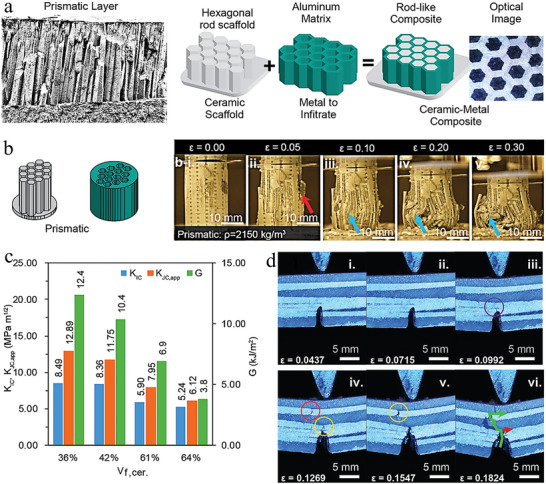
The strengthening and toughening mechanism of composite inspired by nacre's prismatic layer. a) A rod‐shaped ceramic–metal composite inspired by the prismatic layer of the freshwater clam shell. b) Compression behavior of columnar ceramic–metal composites. c) Toughness of initiation fracture (*K*_IC_), fracture toughness of crack propagation (*K*_JC,app_) and total work of fracture (G). d) Evolution of crack propagation for samples with 61% ceramic filled specimens (*V*_f_,cer). Reproduced with permission.^[^ ^83^ ^]^ Copyright 2022, Wiley‐VCH.

The above is mainly an introduction to columnar architecture in the teeth of humans and animals, but columnar architecture is widespread and found in the tissues of other organisms. Common mollusk shells also have a prismatic layer that is primarily used to resist impact loads and wear. These columnar structures serve as an inspiration for scientists to design composites. Inspired by the columnar structure in the mineralized layer, Khan et al. prepared a Si‐Al bionic composite using additive manufacturing and centrifugal infiltration techniques (**Figure** **8**a).^[^ ^83^ ^]^ First, SiO_2_ ceramic prisms were prepared by 3D printing techniques through stereolithography. After that, the voids of the SiO_2_ ceramic prisms were infiltrated by a centrifugal casting technique with molten aluminum alloy. The compression experiments were performed on the ceramic–metal composite with a columnar structure. The results showed that the good toughness of the metal and the ceramic prisms within the metal matrix provided the composite with the ability to withstand the load and deformation (Figure 8b). As the deformation increases, the ceramic prisms first reach the value of failure stress. However, the composite has a good damage tolerance because the failed ceramic prisms are wrapped by the metal matrix and cannot be detached. Additionally, the toughening mechanism of this columnar ceramic–metal composite can be summarized as plastic deformation of the metal matrix, movement limitation of the ceramic prisms, and interface‐induced crack deflection and bridging. As the volume fraction occupied by the ceramic prisms increases, the toughness of crack initiation (*K*_IC_), fracture toughness of crack propagation (*K*_JC,app_) and total work of fracture (*G*) all decrease (Figure 8c). These indicate that as the volume fraction of the ductile metal phase becomes smaller, the effect of the structure on the toughness of the composite decreases. The bending tests of the 61vol%–ceramic composites demonstrated the behavior of crack propagation (Figure 8d). During bending, the presence of defects in the composite itself leads to additional cracking. Crack propagation from the ceramic phase to the metal phase is deflected at the interface until the next defect is encountered, when the crack begins to continue along the defect. Therefore, we found that the inherent defects in the composite can seriously affect the crack initiation and propagation and further affect the various properties of the composite.

In a word, the excellent mechanical properties of the columnar architecture can be attributed to the combination of hard and soft phases. The structure of the hard rod provides strength, while the soft matrix provides excellent fracture toughness. Cracks typically propagate in a direction parallel to the prism, as well as deflecting and bridging in the soft matrix. The deflection and bridging of cracks improve the fracture strain of the composite. Therefore, the hard prism makes the material have better impact and wear resistance, while the soft matrix makes it have the ability to resist fatigue loads. The soft phase provides a buffer layer, which can not only optimize the load transfer, but also absorb energy. Therefore, the combination of the hard phase and soft phase consumes a lot of energy and reduces the initiation and deflection of cracks. However, the development of this architecture is limited by the preparation technology, and it is difficult to control the precision and assembly of the structure at the micro level. Most of the columnar architectures in biomaterials have multiple length scales (atomic, micrometer, and nanometer), which is a challenge for the preparation and assembly of composite materials. Especially for ceramic–metal materials, the brittleness of ceramic prisms and the penetration technology of metals have brought many uncertainties to the preparation of columnar architectures. Here, the columnar architecture is mainly applicable to composite materials that need to improve strength, toughness, wear resistance, and impact resistance, but it is not all.

## 5 Network Architecture

**Figure 9:**
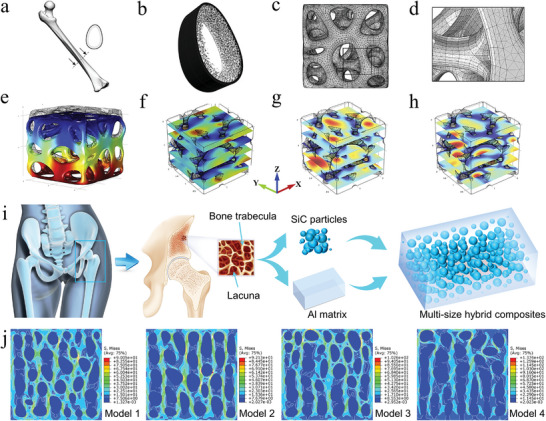
Composite material with a network architecture inspired by the microstructure of bone. a–e) A kind of 3D porous interconnected network model of bone, the finite element modeling, and mechanical properties of the network scaffold model. f–h) The hydrodynamic calculation of the network scaffold was carried out according to the X, Y, and Z axes. a–h) Reproduced with permission.^[^ ^209^ ^]^ Copyright 2016, Elsevier Ltd. i) Inspired by the microstructure of human cancellous bone, a multisize hybrid composite model was established. j) Finite element simulations of stress distribution of cancellous bone under compression. i,j) Reproduced with permission.^[^ ^210^ ^]^ Copyright 2021, Elsevier Ltd.

The network architecture is mainly a porous network structure, and the pores can be optionally filled or unfilled with the material. The composites with this architecture have attracted the interest of engineering and material scientists because of their stable mechanical properties. There are many network architectures in nature, and common ones include bones, glass sponges, porcupine quills, etc.^[^ ^203^, ^204^, ^205^, ^206^, ^207^, ^208^ ^]^ Bone is composed of network trabeculae and bone marrow, and the distributions of trabeculae and bone marrow are used to meet the maximum load and density of the bone. Therefore, microstructures with high mechanical strength for bone can provide inspiration for the preparation of bionic scaffolds and composites. Gómez et al. developed a porous network architecture model of the bone, which can define different porosity gradients in different directions (**Figure** **9**a,b).^[^ ^209^ ^]^ The 3D model of the bone was meshed and simulated for compression experiments, and the material properties were chosen as the most commonly used bone tissue engineering material poly (d,l‐lactide) (Figure 9c–e). The results of compression experiments showed that an increase in the number of nucleation points of the bone‐like porous scaffold leads to an increase in isotropy. Furthermore, by fixing the trabecular thickness or trabecular diameter, a suitable bone‐like porous scaffold can be created. Fluid flow simulations were performed along the x, y, and z axes for tissue models with the bone‐like scaffold removed (Figure 9f–h). The results showed that the computational mechanics and fluid properties of the model were mainly influenced by the total porosity and surface area of the bone. In addition, the technique combined with additive manufacturing technology can also prepare different types of network‐like architectures of scaffolds, which is a general modeling method for network‐like scaffolds.

The bone microstructure with high strength, high toughness, high density, and uniform stress distribution is also applied in the field of particle‐reinforced composites. This is different from the bone‐like porous scaffold described above. Here, the composite material is designed based on the characteristics of the network structure of the cancellous bone, soft phase (bone marrow), and hard phase (bone trabecula). Dai et al. proposed a biomimetic multisize particle‐reinforced composite model by comparing the reinforcing particles to bone marrow and the metal matrix between the reinforcing particles to bone trabeculae (Figure 9i).^[^ ^210^ ^]^ By simulating the compression test for the microstructure of cancellous bone, the high‐stress region is mainly distributed on the bone trabeculae, which therefore bear the majority of the load and homogenize the stress (Figure 9j). However, this multisize composite model replaces the bone trabeculae with a softer matrix that can serve to increase the toughness. Moreover, the dense packing of multisize reinforcing particles instead of bone marrow can result in a denser composite and increase the strength. When the content and size of the reinforcing particles are selected according to the GaussAmp function, the composite has a higher density and a better stress dispersion effect. This bionic multisize particle‐reinforced composite not only improves the strength and plasticity but also has better processability.

**Figure 10:**
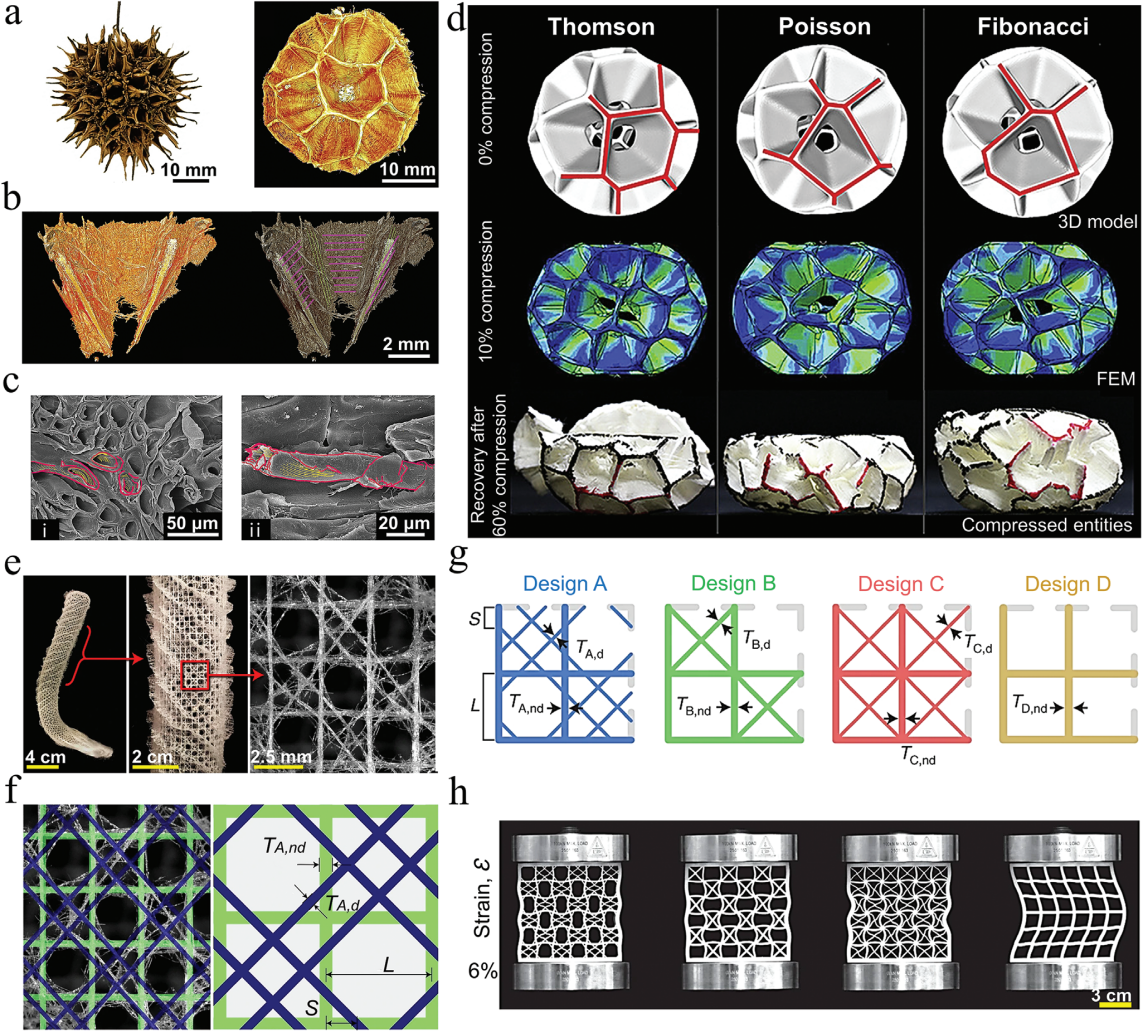
Design of network architecture inspired by Liquidambar formosana and hexactinellid sponge. a,b) The fruit framework of Liquidambar formosana, as well as the single inflorescence unit in the framework and the radial (green line) and parallel (pink line) oriented fibers in the ovary wall. c) SEM images of lignocellulosic‐tubular bundles at the center of the intersection region (i) and the wall region (ii). d) At 0% deformation, 3D spherical models of Thomson, Poisson, and Fibonacci. At 5% deformation, the stress of the Thomson model is concentrated on the junction of the walls, and the other models have spread to the cell walls. At 60% deformation, only the Fibonacci model possessed the recovery capability. a–d) Reproduced with permission.^[^ ^216^ ^]^ Copyright 2020, Elsevier Ltd. e,f) The skeleton structure of the hexactinellid sponge (*Euplectella aspergillum*) contains regular lattice‐like structure, and an ideal truss model is constructed on the skeleton structure. g,h) Schematic diagrams of designs A–D and corresponding mechanical deformation snapshot of 3D‐printed models (under 6% applied strain). e–h) Reproduced with permission.^[^ ^218^ ^]^ Copyright 2021, Springer Nature.

The above mainly introduces the network architecture inspired by the structure of human bone. However, the network architecture is also widespread in plants, which can make the wood materials tough and strong. The lignified network architectures are commonly found in plants such as bamboo, wood, and fruit shells, which are lightweight and have high compressive strength.^[^ ^211^, ^212^, ^213^, ^214^, ^215^ ^]^ Such materials have been used as construction materials since ancient times. Take the example of the lignified maple fruit, as the fruit matures, the ovary wall lignifies and forms a framework that supports the entire fruit (**Figure** **10**a).^[^ ^216^ ^]^ The lignified ovary wall consists of a network of fibers. Most of the fibers are aligned parallel (highlighted with pink lines), while the fibers in crossed areas are aligned radially (highlighted with green lines) (Figure 10b). The cellulosic protofibrils form the walls of the tubular fibers. The hollow fiber bundles in the cross‐center region radiate outward. The fiber bundles in the wall region are aligned tangentially to the cell wall (Figure 10c). Inspired by the framework of maple fruit, Tung et al. proposed three spherical models (Thomson, Poisson, and Fibonacci) and performed compression experiments and simulations (Figure 10d). At 10% deformation, the stress distribution in each chamber of the Thomson model is almost the same, while the different chambers of the Poisson and Fibonacci models have different stress distributions. At 60% deformation, the Thomson model breaks into two parts, the Poisson model has a complete collapse of the upper part, while the Fibonacci model has less damage to the chambers and has the ability to recover after unloading. These differences in behavior may have something to do with the architecture of the three models. The structural symmetry of the Thomson model allows the stresses to be dispersed in the middle region of the model, causing the stresses to reach an upper limit in the middle region and failure of the entire structure occurs. The Poisson model has randomly distributed and irregularly shaped chambers. This causes stresses to be concentrated in some of the sharp walls, which can easily lead to crack initiation and rapid crack propagation. The Fibonacci model has a spiral distribution of chambers, which can avoid continuous deformation and fracture of the structure. As a result, we found that the special structure of the Fibonacci model has higher strength and toughness, and has good recovery after unloading. This lignified network architecture is inspiring in the design of new lightweight, deformation‐resistant and high‐strength composites.

The lattice structure is different from the above structure. It is a geometric network architecture formed by the topology of the unit structure, and the architecture is regular and geometric. Robust lattice structures have great potential for the design of advanced structural materials. Among the wide range of design inspirations for lattice structures, glass sponges have been favored by architects and material scientists for their hard texture and rich geometric network skeleton. Inspired by the network structure, researchers designed many famous buildings, such as the Eiffel Tower.^[^ ^217^ ^]^ Fernandes et al. compared the skeletal structure of the sponge with a 2D square lattice to illustrate the excellent mechanical properties of the sponge skeleton.^[^ ^218^ ^]^ The skeleton of the sponge consists of a network‐like architecture of siliceous fine needles, where the square lattice is reinforced by two sets of crossed pairs of diagonal pillars (Figure 10e,f). Inspired by this network skeleton, the *A*‐structure was designed. In addition, the 2D square lattices compared to the A‐structure are the B‐structure, C‐structure and D‐structure (Figure 10g). Uniaxial compression tests were performed on these four designs of the lattice frame, and the A‐structure was able to carry the highest load and had a higher effective critical flexural stress than the other designs (Figure 10h). From the above analysis, this sponge skeleton structure has high bending resistance under uniaxial compression and can withstand higher loads than the other lattice structures. Therefore, this network‐like architecture of the sponge‐like skeleton can be used for the preparation of high‐performance load‐bearing structures, structure materials, and medical implants.

**Figure 11:**
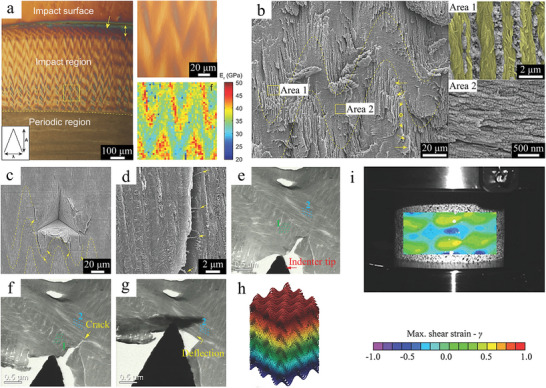
Network architecture inspired by the impact region in mantis shrimp's dactyl club. a) The microstructure of the impact region, the higher‐magnification differential interference contrast image, and high‐resolution nanoindentation map of the region marked in the impact region. b) The compacted helicoidal structure of the herringbone pattern in the impact area. Dashed lines correspond to in‐plane and out of plane fiber orientations. The arrow indicates the local fiber direction. Mineralized fibers oriented out of the plane of the page (area 1) and in the plane of the page (area 2). The out of plane‐oriented fibers show an underlying network of porous canal tubules (yellow) arranged perpendicular to the dactyl‐club surface. c) Indentation of 1000 mN peak load in the impact area. d) Fiber bridging at the indentation edge (yellow arrow). e–g) Progressive stage of loading in the sectioned area of the bulk impact zone. The local microstructure consists of overlapping fiber bundles (green dashed line 1 and blue dashed line 2) oriented perpendicular to each other. These two fiber bundles correspond to the rotating and the pore fibers of the herringbone structure. h) Geometry and fiber orientation of herringbone structure. i) Herringbone structure of the 3D‐printed samples at a strain of 0.1. Reproduced with permission.^[^ ^91^ ^]^ Copyright 2016, Wiley‐VCH.

The regular and geometric network architecture exists not only in the above glass sponge, but also in the mantis shrimp. The architectures of the impact surface and the periodic region of the dactyl club of the mantis shrimp have been described in detail in the previous sections. Here, we present the architecture of the impact region located between the impact surface and the periodic region. The triangular wave pattern can be clearly seen in the cross‐section of the impact region, as well as the elastic modulus of the impact region between 30 and 45 GPa with a herringbone distribution (**Figure** **11**a).^[^ ^91^ ^]^ Further observation of the impact region by SEM revealed that the mineralized chitin fibers were arranged in a spiral pattern and formed a herringbone pattern in the cross‐section. Outside the plane oriented perpendicular to the fracture plane, the mineralized fibers were distributed in vertical fiber pore tubes. In the plane oriented parallel to the fracture plane, the mineralized fibers have a diameter of 49 ± 13 nm (Figure 11b). In fact, the pore tubes not only act as a channel for material transport, but also play a role in strengthening the toughness and strength of the entire structure.

The toughening mechanism of this herringbone network architecture can be found in the nanoindentation images of the impacted region. Most of the cracks exhibit a cessation of propagation in the transition zone between in‐plane and out‐of‐plane. In fact, a small number of cracks were also observed in the out‐of‐plane region, suggesting that the cracks twist with the orientation of the mineralized fibers. The twisting of the cracks is a good form of toughening, as opposed to a simple 90° deflection. Additionally, bridged fibers were observed on the crack surface, and these fibers provided further toughening by releasing the crack tip stress (Figure 11c,d). To elucidate the in‐situ fracture and toughening mechanisms in the impact region, quasi‐static load micro‐indentation tests were performed on the cross‐section of the impact region (Figure 11e–g). First, cracks formed at the prefabricated notch and continued to propagate parallel to the direction of the mineralized fibers within the face (highlighted by the green dashed line). When the crack hits the mineralized fibers within the fiber aperture (highlighted by the blue dashed line), the crack deflects 90° leading to material failure. Moreover, the results of compression experiments and finite element simulations of the herringbone network architecture suggest that redistribution of stress can be translated into redistribution of damage, preventing the rapid failure of partial material (Figure 11h,i). These analyses may provide new insights into changing the fiber structure of the herringbone network architecture to increase the toughness and stiffness of the composites, which can be used to prepare composites that can withstand high strains and high damage tolerances.

From the network architecture presented above, it is clear that the supporting role of bone trabeculae, fibers, and lattice structure makes the composite material have high compressive strength. For the porous network structure, the design and preparation of porous structures are emphasized. The design of porous structure includes the design of gradient, distribution, and shape, which are closely related to the stress distribution, compressive strength, energy absorption, and recovery rate of the composites. However, the microscopic preparation of porous network structures is difficult to control precisely, and the techniques that need to be addressed include structural topology, dimensional control, and structural interconnection. Besides, the material selection of porous structures and matrix is also noteworthy, which indirectly affects the interface bonding and mechanical matching. For the net‐like lattice structure, the compressive strength and mechanical stability of the lattice structure can be fully exploited by the design of gradient, hierarchical and multiple length scales. However, the improvement of mechanical properties requires further exploration of methods to increase the toughness based on mimicking the lattice structure of metal atoms. Increasing the toughness of the lattice structure is helpful in preventing catastrophic damage caused by localized high stresses. For the net‐like fiber structure, the difficulty lies in the design and assembly of the net‐like fibers. The combination of a 3D braided nanofiber network and matrix can resist shear and tensile stresses, and effectively improve the toughness. However, the assembly of nanofibers is difficult to control precisely, which is detrimental to the suppression of crack propagation and stress distribution.

In conclusion, the design of network architecture is developing from the random distribution of network reinforcement to the specific distribution of careful design, which is different from the traditional network structure composites. For most composites, crack deflection, bridging, and interfacial debonding are the primary toughening mechanisms. When the crack propagates in the network architecture composite, the crack tip is subjected to the mixed action of tensile stress and shear stress, which is mainly affected by the network structure and the interface adhesion. In addition to the deflection of the crack, the bridging of the crack and the interfacial debonding also absorb a large amount of energy. Therefore, the network architecture with specific distribution can effectively limit the stress concentration and thus delay the initiation and propagation of cracks. The network architecture of biomaterials is carefully designed. Inspired by this, the network architecture with specific distributed can also achieve some functionality. Moreover, the network structure with specific distributed is conducive to the evaluation of the comprehensive performance of the composite. Here, the network architecture is mainly aimed at composite materials that need to improve strength, toughness, energy absorption, recoverability, and impact resistance, but the applications do not stop there.

## 6 Honeycomb Architecture

**Figure 12:**
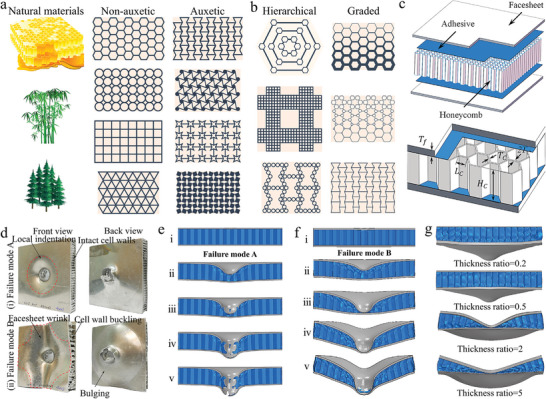
Classification of honeycomb structure inspired by natural materials, and failure mechanisms of hexagonal honeycomb architecture. a) Honeycomb structures with nonauxetic and auxetic inspired by natural materials. b) Two typical hierarchical and graded honeycomb structures. c) Structure and parameters of honeycomb core sandwich. d) Two typical failure modes of sandwich panels with honeycomb core after impact tests. e,f) Failure process of the sandwich panel in failure modes A and B under impact of 381.7 J. g) Deformation characteristics of sandwich panels with different thickness ratios. Reproduced with permission.^[^ ^235^ ^]^ Copyright 2021, Elsevier Ltd.

The elaborate honeycomb architecture is an engineering marvel created by honeybees. Typically, the honeycomb consists of equal‐sized cells, each surrounded by other cells and separated by wax walls.^[^ ^219^, ^220^, ^221^ ^]^ Excitingly, the honeycomb architecture has excellent properties such as lightweight, high strength, sound, and thermal insulation.^[^ ^222^, ^223^, ^224^, ^225^ ^]^ These properties have attracted widespread interest from structural and materials scientists and have led to a wide range of applications for the honeycomb architecture. Furthermore, honeycomb architectures have also been found in the wood of bamboo and spruce, which demonstrates that nature is an excellent structural scientist.^[^ ^226^, ^227^, ^228^ ^]^ Here, we divide the honeycomb architecture into two basic architectures: non‐auxetic honeycomb and auxetic honeycomb. In addition, scientists have developed two typical architectures of hierarchical and graded based on these two architectures (**Figure** **12**a,b). Currently, the honeycomb architecture is developing toward the multiscale, multifunction, and multifield direction.

### 6.1 Nonauxetic Honeycomb

Nonauxetic honeycomb architectures are common in daily life. They are mainly formed by hexagonal, circular, square, and triangular cell topologies.^[^ ^229^, ^230^, ^231^, ^232^, ^233^, ^234^ ^]^ Since the hexagonal honeycomb architecture is the most similar to the structure of a natural honeycomb, the material with hexagonal honeycomb architecture is the most widely studied. Sun et al. conducted a comprehensive study on the structural parameters of sandwich panels with a hexagonal honeycomb core through low‐speed impact tests.^[^ ^235^ ^]^ The detailed structure of the honeycomb core sandwich panel is shown in Figure 12c, with the thickness of the aluminum alloy panel as *T*_f_, the height of the hexagonal honeycomb core as *H*_c_, the hole size as *L*_c_, and the thickness of the hole wall as *T*_c_. The results of the impact tests indicate that the failure modes of the honeycomb sandwich panel include local indentation on both the front and rear panels (failure mode A) and the overall wrinkling of the front panel and local bulging of the rear panel (failure mode B) (Figure 12d). Failure mode A tends to occur in honeycomb sandwich panels with thin panels and stiff cells, while failure mode *B* has the opposite situation. From the results of the finite element simulations, it can be seen that failure mode *A* has five different characteristic stages (Figure 12e). In stage I, the sandwich panel is in elastic deformation and the contact force increases with the increase of displacement. In stage II, the sandwich panel starts to deform plastically, where the honeycomb cell's wall starts to deform in a progressive folding manner from below the deformation area of the front panel. In stage III, the contact force decreases as the front panel breaks and the gap expands. In stage IV, the rear panel resists the impact force causing the contact force to increase again. In stage V, the contact force decreases again as the impactor penetrates the rear panel.

Similarly, failure mode B has five different characteristic phases (Figure 12f). In stages I and II, the situation is the same as in failure mode A. However, in stage III, the contact force decreases slightly and the slope is smaller. In stage IV, the front panel is squeezed into contact with the rear panel resulting in a second increase in contact force. In stage V, the contact force decreases as the displacement increases, as well as the front panel and rear panel gradually break down and fail. For failure mode A, the maximum deformation of the sandwich panel slowly decreases as the thickness of the front panel decreases, while the peak load rises. Instead, the values of maximum deformation and peak load for the sandwich panel become larger as the thickness of the front panel increases in failure mode B. This indicates that the deformation states and failure modes of the sandwich panel depend on the thickness ratio of the front and rear panels. As shown in Figure 12g, the failure mode of the sandwich panel gradually transitions from mode A to mode B as the thickness ratio increases. The above analysis illustrates that the failure mode of the sandwich structure depends largely on the panel thickness, cell size, and wall thickness. Specifically, the height of the honeycomb cell has little effect on the failure mechanism, while the higher density of the honeycomb cell (thick wall or small size) tends to lead to failure mode A. These analyses of structural parameters and failure modes of honeycomb architectures contribute to a better understanding of the potential relationship between structure and performance, which is essential for the preparation of advanced materials with high strength, good energy absorption capacity, and structural stability.

**Figure 13:**
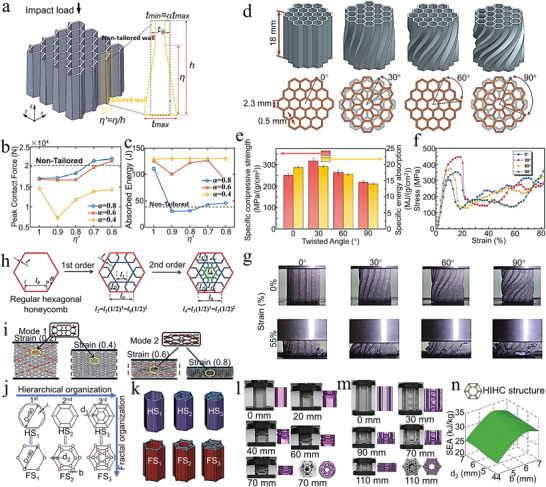
Structure designs and failure mechanisms of graded, twisted, and hierarchical honeycomb architectures. a) Schematic diagram of bilinearly graded honeycomb structure. b,c) As a function of the normalized taper length *η′*, the peak contact force and absorbed energy are obtained by selecting different gradation parameters *α* = {0.8, 0.6, 0.4}. a–c) Reproduced with permission.^[^ ^236^ ^]^ Copyright 2022, Elsevier Ltd. d) Twisted honeycomb structure models with 0°, 30°, 60°, and 90° twist angles. e) Specific compressive strength and specific absorption energy of honeycomb structure under different twist angles. f) Compression load–displacement curves of honeycomb structure under different twist angles. g) Deformation of laser powder bed fusion honeycomb components with different twist angles. d–g) Reproduced with permission.^[^ ^237^ ^]^ Copyright 2020, Elsevier Ltd. h) Self‐similar topological evolution of the first‐order center‐vertex honeycomb and second‐order center‐vertex honeycomb of hexagonal cells added according to the self‐similar hierarchical honeycomb structure. i) Deformation mode of center‐vertex honeycomb, and the representative unit cells have two stable deformation modes. The undeformed vertex hexagonal honeycombs play a support role, and the main deformation in the X‐deformation band is the outermost and innermost hexagonal honeycombs of the representative unit cell (mode 1). The vertex hexagonal honeycombs start to deform (mode 2). h,i) Reproduced with permission.^[^ ^239^ ^]^ Copyright 2021, Elsevier Ltd. j,k) Cross sections of hierarchical and fractal structures, and finite element models of thin‐walled structures. l–n) Deformation modes of hierarchical structures and fractal structure of two layers, and SEA of fractal structures with different geometrical parameters. j–n) Reproduced with permission.^[^ ^244^ ^]^ Copyright 2018, Elsevier Ltd.

In addition to optimizing the basic structural parameters, the reinforced honeycomb architecture also requires innovation based on the natural honeycomb architecture. Honeycomb architecture materials have been a challenge in balancing energy absorption capacity and strength. Usually, honeycomb architectures are composed of honeycomb cells of the same thickness, which is an easy structure to implement in engineering. However, the stresses on honeycomb cells are complex and variable in direction and distribution. Therefore, the honeycomb architecture material with uniform wall thickness needs to be improved in absorbing impact energy. Andrew J et al. designed a geometrically tailored honeycomb with a bilinear wall thickness gradient.^[^ ^236^ ^]^ The structural parameters of the honeycomb are shown in **Figure** **13**a. The *t*_min_ and *t*_max_ denote the minimum and maximum wall thicknesses, and *η* and *h* are the height of the conical base and the total height of the honeycomb. The geometry of this bilinear hierarchical honeycomb can be described by the gradient parameter *α* and the normalized gradient length *η′*. When the gradient parameter *α* is small (*t*_min_ is small), the graded honeycomb has a low initial peak load. In the impact test, the peak contact force increases as *η′* decreases (0.6 ≤ *η′* ≤ 0.9). This is because the design value of *t*_min_ is turned up as *η′* decreases, thus providing a higher resistance to deformation (Figure 13b). Moreover, the energy absorption capacity of the gradient honeycomb increases as the gradient parameter *α* decreases. The energy absorption capacity of the gradient honeycomb increases with increasing the thickness‐gradient, where *α* = 0.4 exhibits sustained resistance to structural collapse for all values of *η′* (Figure 13c). The rupture of this honeycomb with the gradient of bilinear wall thickness starts at the top of the thinner honeycomb wall and destroys the entire honeycomb in a progressive manner of localized folds and crushing from the top to the thicker bottom. Thus, the gradient of wall thickness provides a continuous resistance allowing the honeycomb to have a high total energy absorption capacity. From the above description, it is clear that the honeycomb architecture can be designed to achieve a large energy absorption capacity by adjusting the gradient of wall thickness, while the strength can be improved by adjusting the normalized gradient for length.

In addition to designing the structural parameters and wall thickness of the hexagonal honeycomb, twisting the honeycomb structure at different angles also has certain effects on strength and energy absorption. Lin et al. designed and manufactured honeycomb architectures with twist angles (Figure 13d).^[^ ^237^ ^]^ From the results of the uniaxial compression experiments for the honeycomb structures with twist angles of 0°, 30°, 60° and 90° (Figure 13e–g), it is clear that the highest compressive strength of the honeycomb exhibits 30° (448.7 MPa) > 60° (373.9 MPa) > 0° (354.4 MPa) > 90° (308.2 MPa). Moreover, the value of the specific energy absorption (SEA) is characterized by 30° (19.49 MJ/(g⋅m^3^)) > 0° (19.21 MJ/(g⋅m^3^)) > 60° (17.04 MJ/(g⋅m^3^)) > 90° (14.02 MJ/(g⋅m^3^)). The different twist angles affect the stress distribution in the honeycomb structure, which in turn affects the compressive strength, energy absorption and damage form. For the 0° honeycomb, the honeycomb is compressed into a barrel shape and eventually buckles at the bottom as the strain increases. For the 30° honeycomb, fracture occurs in the middle of the honeycomb at 19% strain and shear damage occurs at 30% strain. For the 60° honeycomb, the crack propagates along the twist direction of the honeycomb and eventually forms the shear collapse. For the 90° honeycomb, cracks appear in the middle of the honeycomb at 10% strain and form shear damage at 18% strain. Therefore, the strength and energy absorption of the honeycomb can be improved by selecting an appropriate twist angle.

In recent years, a typical hierarchical design approach has emerged for honeycomb architectures. The performance improvement of hierarchical honeycomb structures arises from the coupled deformation among the structures. The honeycomb with self‐similar hierarchical structure usually refers to the introduction of small structures with a similar shape to itself at the vertices of the honeycomb geometry.^[^ ^238^ ^]^ Besides, some researchers have also explored hierarchical designs based on honeycomb walls, which will not be further described here. The vertex‐based self‐similar hierarchical honeycomb has been shown to have better stiffness, strength, and energy absorption.^[^ ^239^, ^240^, ^241^, ^242^, ^243^ ^]^ Liang et al. designed a self‐similar hierarchical honeycomb by adding smaller hexagons at the center and vertex of the hexagonal honeycomb (Figure 13h).^[^ ^239^ ^]^ From the deformation mode, it is known that as the strain increases, the honeycomb exhibits the shapes of double‐X, single‐X, and I at the macro level (Figure 13i). Microscopically, when the strains are 0.2 and 0.4, the deformation occurs mainly in the outermost and innermost of the hexagonal structure of the unit cell. At strains of 0.6 and 0.8, the hexagonal structures on the six vertices of the unit cell start to deform. This indicates that the honeycomb has a symmetrically stable and regular deformation mode, which makes the energy absorption process orderly and stable.

The fractal hierarchical honeycomb is an extension of the self‐similar hierarchical honeycomb. In general, the fractal hierarchical honeycomb structures introduce smaller and smaller components with the same shape as themselves. This design is effective in improving mechanical properties. For example, Zhang et al. were inspired by spider webs to design a fractal hierarchical honeycomb, as shown in Figure 13j,k.^[^ ^244^ ^]^ From the damage mode of the two‐layer hierarchical honeycomb, the progressive folding deformation starts uniformly from the top and has a long folding wavelength (Figure 13l). From the damage mode of the two‐layer fractal honeycomb, the honeycomb has very regular and uniform folds, and the wavelength of the folds is very short. Additionally, the folding deformation starts at the bottom. This regular and stable collapse is the expected energy absorption pattern (Figure 13m). Figure 13n depicts the 3D surface of the HIHC structure with different side lengths of sub‐hexagon and connecting segments (b and d_2_). It is emphasized that the value of the connecting distance has a small effect on the SEA, while the side length of the sub‐hexagon has a strong effect on the SEA (increasing and then decreasing). Therefore, the side length of the sub‐hexagon plays an important role in the design of the fractal hierarchical honeycomb for crashworthiness.

**Figure 14:**
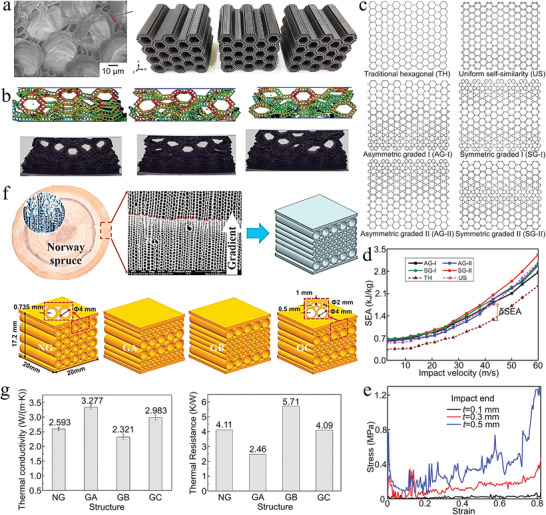
Structure designs of graded‐hierarchical and graded‐circular honeycombs. a,b) Inspired by the cellular structure of bamboo, three bamboo biomorphic structures of BBS‐GS_Low, BBS‐GS_Mid, and BBS‐GS_High were designed. After that, the deformation of these three structures is studied by the finite element method and experiment. Reproduced with permission.^[^ ^245^ ^]^ Copyright 2021, Elsevier Ltd. c) Six geometric architectures of graded fractal honeycombs. d,e) The relationship between SEA and the impact velocity for six kinds of graded fractal honeycombs, and the stress–strain curves of the AG‐II honeycomb with different wall thickness. c–e) Reproduced with permission.^[^ ^246^ ^]^ Copyright 2021, Elsevier Ltd. f) No gradient structure (NG) and gradient structure A‐C (GA‐GC) were designed according to the structure of Norway spruce. g) The measured thermal conductivity and the thermal resistance (quarter of top part) of the Ti6Al4V component subjected to selective laser melting treatment. f,g) Reproduced with permission.^[^ ^247^ ^]^ Copyright 2019, Elsevier Ltd.

In addition to the hierarchical structures mentioned above, a functional gradient honeycomb is also a way to improve strength, stiffness, and energy absorption. Currently, combining gradient and hierarchical structures to design honeycomb architectures is considered to have potential. Ufodike et al. designed a gradient bamboo biomorphic structure (BBS‐GS) inspired by the cell wall structure of bamboo.^[^ ^245^ ^]^ Specifically, the gradient bamboo biomorphic embodies the introduction of gradient wall thickness in the honeycomb structure as well as the introduction of small square and triangular cavities in the honeycomb walls. Based on this idea, three honeycomb structures were designed for BBS‐GS_Low (layer difference: 0.25 mm), BBS‐GS_Mid (layer difference: 0.325 mm) and BBS‐GS_High (layer difference: 0.4 mm) (**Figure** **14**a). From the uniaxial compression morphology of the three honeycomb structures, the design of BBS‐GS allows the structure to improve energy absorption during failure by progressive deformation (Figure 14b). Thus, the gradient structure is effective in absorbing energy. In addition to setting the gradient on wall thickness, setting the gradient on the structure is also a good strategy. The gradient of the honeycomb structure is a combination of honeycomb with different shapes and structures, which provides a new idea for honeycomb materials under specific conditions. Further, Liu et al. combined the gradient with fractal self‐similarity to propose a honeycomb structure with symmetric and asymmetric gradients (Figure 14c).^[^ ^246^ ^]^ The results of the impact experiments showed that the specific energy absorption of all graded fractal honeycombs was significantly increased compared to the TH honeycombs. Moreover, the lower impact velocity favors the SG‐I honeycomb with better energy absorption performance, while the SG‐II honeycomb performs better at high impact velocity (Figure 14d). By observing the effect of different wall thicknesses on the stress‐strain of the AG‐II honeycomb, the stress increased with the thickening of the honeycomb wall, especially the stress at the impact end and distal end increased significantly (Figure 14e). These results can provide a reference for the design of energy absorbers at different impact velocities and wall thicknesses.

Honeycomb materials can be used not only for energy absorption, but also for thermal protection. Inspired by the structure of the Norway spruce trunk, Lin et al. proposed a gradient honeycomb structure for thermal insulation.^[^ ^247^ ^]^ Four different structures of honeycomb are shown in Figure 14f, the gradient and nongradient honeycombs were designed in the sandwich panel. By measuring the thermal conductivity and thermal resistance of the different structures, it can be found that the GB structure possesses the lowest thermal conductivity and the highest thermal resistance (Figure 14g). However, the thermal resistance of GA and GC structures is lower than that of the NG structure, which indicates that the gradient structure does not guarantee good thermal insulation of the honeycomb material.

### 6.2 Auxetic Honeycomb

**Figure 15:**
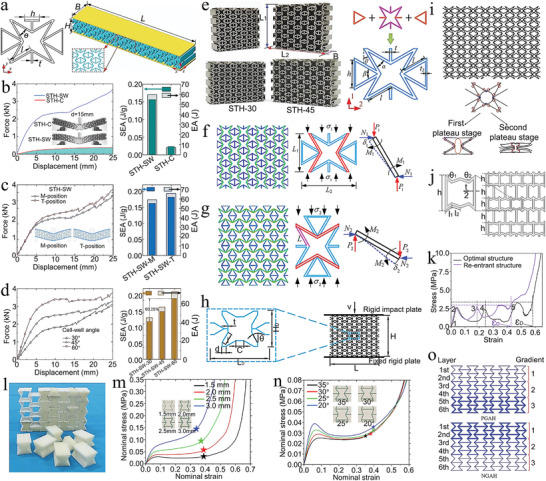
Structure designs and strengthening mechanisms of auxetic honeycomb architectures. a) Geometrical architecture of the sandwich beams with star‐triangular honeycomb core structure. b) Mechanical properties of STH‐SW and STH‐C under three‐point bending tests. c,d) Force–displacement curves and energy absorption capacities of sandwich beams with different indenter positions and cell‐wall angles. a–d) Reproduced with permission.^[^ ^255^ ^]^ Copyright 2021, Elsevier Ltd. e) Geometry structure and samples of star‐triangle auxetic honeycombs. f,g) The Von Mises stress in 1‐direction and 2‐direction of star‐triangular honeycomb. e–g) Reproduced with permission.^[^ ^256^ ^]^ Copyright 2021, Elsevier Ltd. h) Unit cell and geometric parameters of the bidirectional re‐entrant honeycomb. Reproduced with permission.^[^ ^257^ ^]^ Copyright 2021, Elsevier Ltd. i) Deformation mechanism of the representative unit of the star‐circle honeycomb. Reproduced with permission.^[^ ^258^ ^]^ Copyright 2020, Elsevier Ltd. j,k) Schematic diagram of the modified re‐entrant honeycomb structure, and the stress–strain curves for regular re‐entrant honeycomb and optimal structure. Reproduced with permission.^[^ ^259^ ^]^ Copyright 2022, Elsevier Ltd. l–n) Samples of foam‐filled honeycomb, and the stress–strain curves of slow‐recovery foam filled with different cell wall thicknesses and cell angles re‐entering the honeycomb. Reproduced with permission.^[^ ^260^ ^]^ Copyright 2022, Elsevier Ltd. o) Schematic diagram of the positive gradient re‐entrant honeycomb (PGAH) and negative gradient re‐entrant honeycomb (NGAH). Reproduced with permission.^[^ ^261^ ^]^ Copyright 2021, Elsevier Ltd.

Auxetic honeycomb has been focused on due to its unique negative Poisson's ratio properties and mechanical properties. Common auxetic structures include star, rotated square, re‐entrant arrow, re‐entrant hexagon, and chiral (“anti‐trichiral”).^[^ ^248^, ^249^, ^250^, ^251^, ^252^, ^253^, ^254^ ^]^ The carefully engineered honeycomb with negative Poisson's ratio has excellent fracture toughness, indentation resistance, shear modulus, and energy absorption. Zhao et al. designed a sandwich beam (STH‐SW) consisting of a star‐triangle honeycomb core (STH‐C) and investigated the bending performance by a three‐point bending test.^[^ ^255^ ^]^ The honeycomb cell placed in the middle of the sandwich beam was formed by the star‐triangle structure topology (**Figure** **15**a). The results of the three‐point bending test showed that the compressive force and energy absorption capacity of STH‐SW at the same indenter position were more than five times that of STH‐C. At different indenter positions, the compression force and energy absorption capacity of the sandwich beam at the T position were significantly higher than those at the M position. In addition, the load and energy absorption capacity of STH‐SW increased with the increase of the wall angle for the unit cell. In summary, panel thickness, indenter position and wall angle have effects on the load and energy absorption capacity of the sandwich beam (Figure 15b–d). To further clarify the deformation mechanism of STH, the samples in Figure 15e were fabricated using additive manufacturing technology.^[^ ^256^ ^]^ By assuming that the STH does not have the effect of boundary and the changes of geometric structure, the deformation mechanism of the typical unit cell structure was established. As shown in Figure 15f, under the stress *σ*_1_, the elastic modulus *E*_1_ and Poisson's ratio *v*_12_ of STH are

(2) $$E_{1}=\frac{E_{s}t^{3}\cos\theta }{\begin{array}{c} \left(2\cos\theta -\sin\theta \right)\left[l-\frac{t}{2}\left(\frac{1}{\tan\alpha /2}-\frac{1}{\tan\alpha }\right)\right]\\ \;\times \left\{\;{\left[l-\frac{t}{2}\left(\frac{1}{\tan\alpha /2}-\frac{1}{\tan\alpha }\right)\right]}^{2}\sin^{2}\theta +t^{2}\cos^{2}\theta \;\right\} \end{array}}$$

(3) $$\begin{array}{c} v_{12}=-\frac{\cos^{2}\theta \sin\theta \;\left\{\;{\left[l\;-\;\frac{t}{2}\left(\frac{1}{\tan\alpha /2}\;-\;\frac{1}{\tan\alpha }\right)\right]}^{2}\;-\;t^{2}\;\right\}}{\left(2\cos\theta \;-\;\sin\theta \right)\;\left\{\;{\left[l\;-\;\frac{t}{2}\left(\frac{1}{\tan\alpha /2}\;-\;\frac{1}{\tan\alpha }\right)\right]}^{2}\sin^{2}\theta \;+\;t^{2}\cos^{2}\theta \;\right\}} \end{array}$$

where *E*_s_ is the bending stiffness of STH. *α* is the angle of the unit wall. *t* is the thickness of the recessed unit wall. *θ* is the inclination angle of the recessed unit wall.

As shown in Figure 15g, under the stress *σ_2_*, the modulus of elasticity *E*_2_ and Poisson's ratio *v*_21_ of STH are

(4) $$E_{2}=\frac{E_{s}t^{3}\left(2\cos\theta -\sin\theta \right)}{\begin{array}{c} \left[l-\frac{t}{2}\left(\frac{1}{\tan\alpha /2}-\frac{1}{\tan\alpha }\right)\right]\\ \;\times \left\{\;{\left[l-\frac{t}{2}\left(\frac{1}{\tan\alpha /2}-\frac{1}{\tan\alpha }\right)\right]}^{2}\cos^{2}\theta +t^{2}\sin^{2}\theta \;\right\}\cos\theta \end{array}}$$

(5) $$v_{21}=-\frac{\sin\theta \left(2\cos\theta -\sin\theta \right)\;\left\{\;{\left[l-\frac{t}{2}\left(\frac{1}{\tan\alpha /2}-\frac{1}{\tan\alpha }\right)\right]}^{2}-t^{2}\;\right\}}{{\left[l-\frac{t}{2}\left(\frac{1}{\tan\alpha /2}-\frac{1}{\tan\alpha }\right)\right]}^{2}\cos^{2}\theta +t^{2}\sin^{2}\theta }$$

The above researches comprehensively describe the deformation mechanism of star honeycomb, and the influence of structural parameters and indenter position on the load capacity and energy absorption capacity. However, these studies of star honeycombs do not involve the modification of structure and shape. Thereafter, An et al. modified the star‐shaped honeycomb and designed a bidirectional re‐entrant honeycomb as shown in Figure 15h.^[^ ^257^ ^]^ The bidirectional re‐entrant honeycomb has a better energy absorption capacity than the conventional star‐shaped honeycomb. Moreover, the negative Poisson's ratio effect of the bidirectional re‐entrant honeycomb is more obvious under low‐speed compression. Moreover, Lu et al. replaced the horizontal walls of the star honeycomb with double inclined walls and designed circular walls inside the star (Figure 15i).^[^ ^258^ ^]^ From the deformation process of this honeycomb structure, the cross‐inclined walls improve the stability of the structure deformation. Furthermore, the deformation between the thin‐walled circle and the inclined wall makes the honeycomb structure appear more like plastic hinges, which improves the energy absorption capacity and impact resistance.

In addition to the star honeycomb mentioned above, the re‐entrant hexagonal honeycomb is also a typical auxetic honeycomb. In recent years, scientists have carried out innovative research on the basis of the re‐entrant hexagonal honeycomb. Choudhry et al. designed the inclined struts of the re‐entrant hexagonal honeycomb in a “*W*” shape and the length is the same as the original length (Figure 15j).^[^ ^259^ ^]^ The stress–strain curve of the modified re‐entrant hexagonal honeycomb shows that the value of stress increases and then decreases with the collapse of the honeycomb layers (Figure 15k). When all the honeycomb layers collapse and densify, the stress starts to increase continuously. This improvement in the energy absorption capacity of the modified re‐entrant hexagonal honeycomb can be attributed to the addition of low rotational stiffness nodes in the structure. In addition to improving the mechanical properties of the honeycomb by changing the structure, filling the re‐entrant hexagonal honeycomb with a cushioning material is also a good idea. Luo et al. investigated the performance of a re‐entrant hexagonal honeycomb filled with slow‐recovery foam (Figure 15l).^[^ ^260^ ^]^ The wall thickness and angle of the honeycomb had a significant effect on the mechanical properties of the re‐entrant honeycomb filled with slow‐recovery foam. An increase in the wall thickness of the honeycomb cell leads to an increase in the overall stiffness of the structure (Figure 15m). Furthermore, the decrease in the honeycomb angle will lead to an increase in the volume of the filled slow‐recovery foam. This results in an increase in the initial structure stress, platform stress, and load carrying capacity (Figure 15n). Furthermore, the gradient design can also improve the mechanical properties of the re‐entrant hexagonal honeycomb. Shao et al. investigated the effect of positive and negative gradients on the mechanical properties of the re‐entrant hexagonal honeycomb.^[^ ^261^ ^]^ For positive gradient re‐entrant hexagonal honeycomb (PGAH), as the compressive strain increases, the thinnest wall layer starts to shrink and leads to an increase in crushing stress. For the negative gradient re‐entrant hexagonal honeycomb (NGAH), the NGAH has a lower horizontal strain and better energy absorption capacity under high‐speed compression (Figure 15o).

For honeycomb sandwich panels, the mechanical properties and failure modes mainly depend on the panel thickness, honeycomb cell size, wall thickness, and honeycomb cell height. The failure of honeycomb architecture is to destroy the whole honeycomb in a progressive way of local folding and crushing. Therefore, the key to improving the mechanical properties of honeycomb architecture is to improve the stability of structural deformation and make it have the ability to continuously resist deformation. For this reason, fractal hierarchical and gradient honeycombs are designed. This kind of honeycomb architecture has a stable and regular deformation mode, which makes the destruction process orderly and stable, and improves the energy absorption capacity of the honeycomb architecture. To further improve the mechanical properties, composite with auxetic honeycomb structures have been developed. The negative Poisson's ratio effect makes the honeycomb structure have excellent fracture toughness, indentation resistance, and energy absorption capacity.

To sum up, honeycomb architectures with nonauxetic and auxetic are widely used because of their excellent energy absorption capacity and compression resistance. Furthermore, the negative Poisson's ratio effect shown by the auxetic honeycomb makes it more attractive in terms of crashworthiness. Mechanical strengthening of honeycomb structures can be considered from structural parameters, hierarchical, fractal and gradient design. However, the deformation mode and energy absorption capability of honeycomb structures are affected by nonuniform loads. In the design of honeycomb architecture, the stress distribution and failure mode of honeycomb under nonuniform load should be fully considered. Moreover, the gradual deformation of honeycomb materials is the key to improving energy absorption. Therefore, the structural design to control the deformation process of the honeycomb can further improve crashworthiness and energy absorption. Here, the honeycomb architecture is mainly aimed at composite materials that need to improve the lightweight, heat dissipation, load‐bearing capacity, and noise control, but it is not limited to these.

## 7 Multitype Composite Architecture

**Figure 16:**
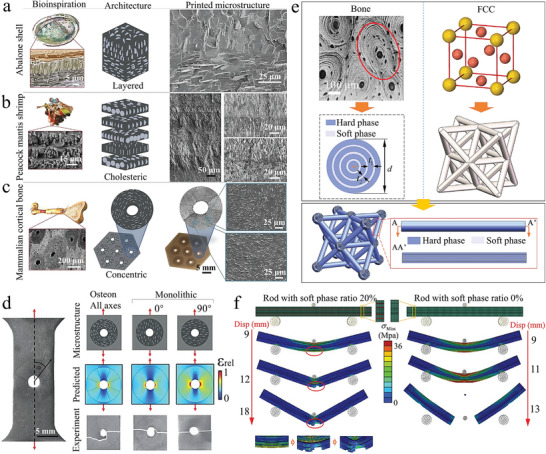
Multitype composite architecture inspired by abalone shell, mantis shrimp and cortical bone. a–c) The bioinspired composites are printed according to the complete microstructural architectures of abalone shell (nacre, prismatic, and periostracum), peacock mantis shrimp (impact surface, impact region, and periodic region) and mammalian cortical bone (bone lamella, Haversian canal, and collagen fiber, etc). d) Structural performance of printed composites with circular defects. a–d) Reproduced with permission.^[^ ^262^ ^]^ Copyright 2015, Springer Nature. e) Composites inspired by face‐centered cubic lattice and bone structure. f) The concentric soft‐hard rods of the three‐point bending simulation. e,f) Reproduced with permission.^[^ ^35^ ^]^ Copyright 2022, Elsevier Ltd.

The tissues with excellent mechanical properties in organisms are mostly formed by the coupling of multitype structures. The coupling of multitype structures overcomes the shortcomings of the single structure, which allows biomimetic structural materials to combine high strength, low density, and high toughness. Therefore, the development of multitype composite architecture is a necessary path to completely imitate biological structures. Martin et al. successfully reproduced the architectures of abalone shell, mantis shrimp's dactyl club, and cortical bone with “3D magnetic printing” (**Figure** **16**a–c).^[^ ^262^ ^]^ The complete architecture of these organisms is a combination of some basic architectures. For example, the architecture of the bionic cortical bone is a porous cylinder composed of ceramic slices and plywood. Multitype structural composites with reinforcements in all axes, 0° and 90° directions were prepared by 3D magnetic printing (Figure 16d). From the finite element simulation results, it is clear that the maximum relative strain occurs at the edges of the circular pores. Furthermore, the cracks of both the osteon structure and the 0° structure showed deflection, while the 90° structure has no deflection. These results indicate that the bionic bone structure has good crack deflection in the circumferential direction, thereby improving the fracture toughness of the material. This structure is similar to rolling the nacre structure into a cylindrical shape with holes, which overcomes the limitation of a single nacre structure in the direction of the force.

Based on the above cortical bone architecture, the bionic cortical bone architecture and lattice structure can be further combined to design multitype composite architectures. Wei et al. proposed a multitype composite architecture by combining the concentric circle structure (inspired by bone) and the face‐centered cubic structure (inspired by crystal structure) (Figure 16e).^[^ ^35^ ^]^ The concentric circle structure consists of the soft phase and hard phase. The concentric circle structure with 20% of the soft phase exhibits better toughness in three‐point bending simulations (Figure 16f). Besides, the energy absorption capability of this structure is about 3.8 times higher than that of the structure which is composed of the hard phase only. This is due to the fact that the presence of the soft phase inhibits crack propagation. In addition, the presence of the face‐centered cubic structure allows the material to undergo three stages of deformation: elastic, bending, and compaction. Therefore, this multitype composite architecture gives the material high strength, high toughness, lightweight, and good energy absorption capability.

**Figure 17:**
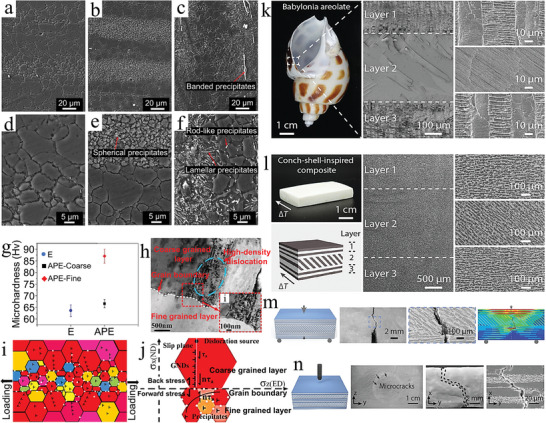
Design and strength‐toughness mechanisms of heterogeneous laminar architectures. a–f) SEM micrographs of a,d) E sample, b,e) APE sample, and c,f) EPA sample. g,h) Microhardness and TEM images of APE samples. i,j) Model diagrams of dislocation accumulation and non‐uniform deformation process in APE samples. a–j) Reproduced with permission.^[^ ^263^ ^]^ Copyright 2021, Elsevier Ltd. k,l) Images of the three macroscopic lamellae of the conch shell and the microstructure of the composite inspired by them. m) Optical images of crack propagation in conch–shell inspired composite with three layers under quasi‐static loading. n) Optical images of crack propagation in conch‐shell inspired composite with five layers under dynamic loading. k–n) Reproduced with permission.^[^ ^264^ ^]^ Copyright 2022, Wiley‐VCH.

The lamellar architecture plays an important role in crack deflection, so the combination of the lamellar architecture and other types of architecture might bring the performance of the structural material to a higher. The lamellar architectures can be combined with homogeneous and inhomogeneous architectures to design multitype composite architectures. Liu et al. fabricated the architecture with heterogeneous and lamellar structure by combining the coarse‐grained and fine‐grained layers (**Figure** **17**a–f).^[^ ^263^ ^]^ For the extruded sample with a homogeneous structure (*E*), partial precipitates were found at the grain boundaries. These precipitates are mainly caused by dynamic precipitation of the precipitated phase and broken coarse grains. For the heterogeneous laminated samples aged before extrusion (APE), the precipitated phases were mainly distributed at the grain boundaries of the fine‐grained layers. For the samples aged before extrusion (EPA), the precipitations show banded, discontinuous and continuous morphologies. These stable precipitations enhance the effect of pinning and hindering dislocations, so that the samples have high strength. Moreover, heterogeneous deformation‐induced (HDI) strengthening and work hardening can also improve mechanical properties. As shown in Figure 17g, the microhardness of the fine‐grained layer of the APE sample is much higher than that of the coarse‐grained layer and the *E* sample. This indicates the presence of heterogeneous strength layers in the APE samples, which creates conditions for geometrically necessary dislocations. Additionally, the geometrically necessary dislocations were mainly found in the coarse‐grained layers near the grain boundaries (Figure 17h). During the tensile deformation of the APE sample, the coarse‐grained layer is first plastically deformed and restrained by the fine‐grained layer due to the higher microhardness of the fine‐grained layer. As the deformation increases, the strain gradient in the coarse‐grained layer is regulated by the accumulation of geometrically necessary dislocations. The back stresses generated in the coarse‐grained layer and the forward stresses generated in the fine‐grained layer comprise the HDI stresses (Figure 17i,j). Therefore, the enhancement of HDI strengthening is attributed to the accumulation of geometrically necessary dislocations at grain boundaries. Moreover, the synergistic deformation between the coarse‐grained and fine‐grained layers leads to work hardening. From the above analysis, we can see that this heterogeneous layer architecture can effectively enhance the strength and ductility of the material.

In addition to combining with homogeneous and inhomogeneous architectures, lamellar architectures can also be combined with aligned structures to design multitype composite architectures. The toughness of the conch shell is much higher than that of the nacre, because it is a composite of three layers with different orientations. This combination of lamellar and aligned structures allows more paths for crack propagation, which improves the fracture toughness of the material. Li et al. prepared a composite material imitating the conch shell using the freeze‐casting method (Figure 17k,l).^[^ ^264^ ^]^ The three‐layer material of the conch‐shell biomimetic composite consisted of thin sheets and polymers with a specific orientation. The combination of thin sheets and polymers is similar to the nacre of the “brick‐and‐mortar” structure. Besides, the first and third layers of the composite are aligned in the same direction, while the second layer in the middle is aligned with a certain angle of deflection. As shown in Figure 17m, the structure of the second layer of the composite caused additional crack deflection, which indicates that the overall toughness of the composite is better than that of the nacre structure. From the results of the drop tower test, it can be found that the biomimetic composite developed only microcracks under the impact of the impactor and did not develop into a catastrophic fracture (Figure 17n). The propagation and deflection of the microcracks in this architecture cause more energy dissipation, which makes it have better impact resistance.

In conclusion, high‐performance and multifunction composites are the goals pursued by scientists. To achieve this goal, the single‐type architecture has been difficult to meet the requirements, while the comprehensive performance of multitype composite architecture is excellent. The multitype composite architecture realizes the high strength, toughness, damage tolerance and energy absorption capacity of composite materials through the coupling of multistructure, multiscale, and multifunction. However, the preparation of multitype composite architecture is challenging, including multiscale manufacturing, orientation adjustment, real‐time raw material switching, and preparation precision. In addition, the design of composite architectures involves the mechanical coupling of multiple types of structures and the optimal selection of structures. Therefore, with the help of simulation technology and machine learning, the theoretical design of composite architectures may be improved.

## 8 Conclusion and Outlook

**Figure 18:**
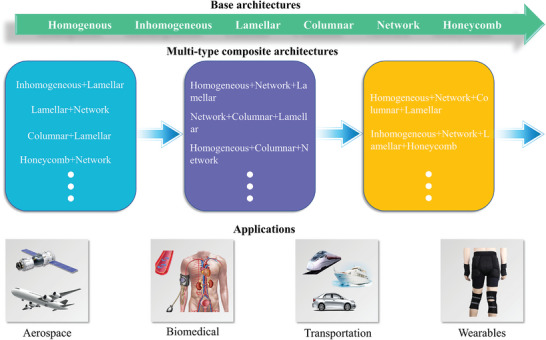
Summarize on the types and applications of architectures. Basic architectures inspired by biomaterials have been developed and applied, and more will be developed in the future. It is noteworthy that with the higher demand for the mechanics and functions of materials, the multitype composite architecture composed of basic architectures is being rapidly developed. Advanced composite materials produced on the basis of these architectures can be used in aerospace, biomedical, transportation, wearable, and other fields.

The design of architecture is expected to strengthen the properties of composites and develop multifunctional advanced materials. In recent years, the preparation and application of structural materials have developed rapidly, but the theoretical design and preparation technology of architecture need to be further improved. Here we discuss and summarize the strengthening mechanism and challenges of basic architecture and multitype composite architecture. The technology roadmap lists the types of architectures and predicts four types of composite architectures composed of four basic architectures (**Figure** **18**). Only by understanding the strengthening mechanism of each architecture can we have a deeper and more comprehensive understanding of the mechanical properties, unique functions, and applicable fields of structural materials. Finally, we present our views on the challenges and opportunities that the design and preparation of structural materials may face, and hope to advance its development in the fields of aerospace, biomedical, transportation, and wearable devices.

The design of traditional architecture has focused on the improvement of strength, toughness, damage tolerance, wear resistance, and some physical properties, such as electrical and thermal conductivity. Although the existing technology has been able to prepare these structural materials, the actual performance is still far below the theoretical performance. The reasons for this result are multiple, but it is mainly due to the low precision of the preparation technology and unreasonable design of the architecture. In recent years, additive manufacturing technology has developed rapidly in the preparation of structural materials. However, for most bionic architecture, we cannot prepare fine structures with the same scale as natural materials. This is mainly because most of the preparation technologies cannot meet the design requirements in terms of accuracy. Besides, the design of microarchitecture is more conducive to improving the performance of materials. For multitype composite architecture, theoretical design is a challenging task. The design of composite architecture is unknown, which is reflected in the need to determine the component material of the overall structure and the special architecture of each structural unit. For example, the multitype composite architecture of the mantis shrimp's dactyl club is divided into three layers in macroscopic dimensions. The outermost layer is a densely packed structure of particles that provides resistance to impact loads. The wavy network structure in the middle layer can play the role of twisting crack. The Bouligand structure of the inner layer plays the role of energy absorption. Therefore, the coupling of some architectures and the preparation of multiscale structures are key technologies that need to be overcome.

Specifically, **Table** **1** compares the strengthening mechanisms, fabrication methods, advantages, and limitations of several typical architectures. For homogeneous and inhomogeneous architectures, the introduction of multiphase components and ultra‐fine structure design has made a breakthrough in the properties of composites. However, in the preparation process, it is difficult to achieve the bonding between different phases, the uniform refinement of the structure, and the control of the ultrafine structure of different phases. It is known that the bonding of strong interfaces and the uniform refinement of grains can make the composites have higher hardness and wear resistance. However, the key to improving toughness is the introduction of soft‐hard phase microstructure and gradient design, which can improve the damage tolerance of composites and further enhance toughness. For laminar and columnar architectures, the combination of laminar and columnar hard phases with the intermediate distribution of soft phases is the key to breaking the paradox of strength and toughness. The redistribution of shear strain between soft and hard phases and the deflection of cracks can effectively improve the toughness and strength of composites. However, assembly and control techniques of microstructure are critical to the development of structural materials. Perfect structural materials need superior assembly technology and preparation technology. Although additive manufacturing has made great progress in precision and speed, commercially feasible 3D printers still cannot meet the requirements in terms of ultra‐microscale and hierarchical scale.

**Table 1:** Comparison of representative strengthening mechanisms, fabrication methods, advantages, and limitations as important factors for typical architectures

Architecture type		Strengthening mechanisms	Typical materials	Fabrication methods	Advantages	Limitations	Refs.
Homogenous and inhomogeneous	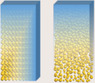	Dislocation, fine‐grain, substructure, solid solution, precipitation, dispersion, second phase, aging, reinforcement body, crack deflection, gradient strengthening	Metal alloy, composite with reinforcement, gradient material	Casting, additive manufacturing, powder metallurgy, in‐situ synthesis	high strength, high toughness, adjustable density, easy preparation	Poor wettability, uncontrollable dispersion state, ultramicro preparation technology	[149, 150, 151, 165, 166, 170]
Lamellar	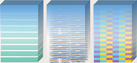	Multiscale, hard‐soft phase, crack deflection and bridging	Nacre, laminated reinforcement composite, soft‐hard layer composite	Freeze casting, sintering, vacuum infiltration, co‐extruding, additive manufacturing, roll bonding	Impact resistance, high strength and toughness, high damage tolerance	Imperfect layer assembly, interface bonding/reaction, micro‐layer dimension control	[187, 188, 189, 192, 193, 194, 195, 196]
Columnar	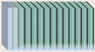	Crack deflection and bridging, multiphase toughening	Abalone's prismatic layer, prisms of enamel, mineralized shell of Chiton teeth	Additive manufacturing, casting, sintering	Impact resistance, fatigue load resistance, high hardness, high elastic modulus	Prismatic alignment, micro size control, assembly technology	[83, 201, 202]
Network	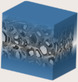	Crack deflection and twist, prevent crack propagation, reinforced truss, microcracking resistance	Cellular materials, lattice structure material, cancellous bone, lignified shell	Additive manufacturing, powder metallurgy, reactive synthesis	High energy absorption, impact resistance, damage resistance, high strength and toughness, lightness	Micro size control, microstructure expansion, preparation process	[91, 209, 210, 216, 218]
Honeycomb	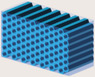	Progressive collapse, plateau stress, densification stage, gradient design, hierarchical design, fractal design, negative Poisson's ratio effect	Nonauxetic honeycomb, auxetic honeycomb	Additive manufacturing, laser powder bed fusion, fused filament fabrication	High energy absorption, High compressive strength, lightness, heat dissipation, vibration reduction, noise reduction	Irrecoverable deformation, directional compressive strength	[235, 236, 237, 238, 239, 244, 245, 246, 247, 255, 256, 257, 258, 259, 260, 261]
Multitype composite	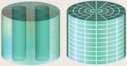	Multiscale, crack deflection, microcracking resistance, prevent crack propagation	Conch shell, composite reinforcement architectures, abalone shell, mantis shrimp's dactyl club	Additive manufacturing, assembly technology	High strength, high toughness, high stiffness, lightness	Difficult preparation, complex design	[35, 262, 263, 264]

Network architecture is the most common in nature. To develop the network architecture, it is necessary to understand the mechanical properties of the network scaffold and the binding mechanism between the scaffold and the matrix. Besides, the preparation of network scaffolds is also a key challenge. Complex network scaffolds require strict control of scaffold shape and void distribution. For the honeycomb architecture, the design of shape and size is the key factor to be considered. Fractal and self‐similar honeycomb architectures have been shown to have excellent strength and energy absorption capacity. The progressive failure of the structure is beneficial to improve the impact resistance. Unfortunately, the mechanical properties of honeycomb structures are quite different in different directions. The nonuniform stress with spatial variation is more common in practice. Therefore, the design of the honeycomb structure should comprehensively consider the stress and failure form of the structure under the action of nonuniform stress. To combine the advantages of all types of architecture, multitype composite architecture was designed. At present, this kind of architecture faces the challenges of multiscale preparation and structural coupling. With the rapid development of structural simulation technology, the coupling performance between different structures will be more intuitive. In addition, additive manufacturing technology has realized the multiscale production from micro to macro, and can be fine‐printed region by region. The progress of these technologies has promoted the development of multitype composite materials.

Looking ahead, the architecture design of advanced materials still faces many opportunities and challenges. In the development process of advanced materials from single‐scale and single‐structure to multiscale and multitype composite structure, a lot of meaningful topics have emerged in architectural design, mechanical simulation, and material preparation. The development of advanced preparation technology, simulation technology, characterization technology, and computer‐aided design has further improved the structure and function design of advanced composites. The progress of preparation technology has enabled us to prepare composite materials at multiple length scales (atomic, nanoscale, and micrometer). Simulation and characterization technologies have deepened our understanding of the relationships among microstructure, overall performance, and function. In addition, computer‐aided technology provides a powerful platform for structural modeling, simulation prediction, and material selection. These technological advances have brought hope for the development of structural materials. To prepare composites with better performance and benefit society, some challenges must be overcome.

First, multiscale design is the key to giving full play to the strengthening effect of architecture and integrating the structural performance of each length scale. The combination of multiple scales can better realize the strength and toughness matching performance, and discard the shortcomings of single length structure. Furthermore, multiple scales have achieved remarkable results in stress distribution, energy dissipation, and crack deflection, and have great potential in preparing composite materials with superior comprehensive properties. Composite materials with complex structures and excellent mechanical properties are created by combining multiple length scales. The toughness and strength of these composites can be changed in a large range by adjusting the size of the reinforcement to achieve adjustable mechanical properties. In addition, some bionic materials with wear resistance, impact resistance and high toughness can be designed by imitating the structures of biomaterials with multiple length scales. However, due to the limitations of the preparation technology, the structure of perfect replica biomaterials still cannot be achieved. Here, we expect that with the development of manufacturing processes (such as 3D printing and self‐assembly), the difficulties in the preparation and assembly of complex multiscale composite materials are expected to be improved.

Second, the design of multitype composite architecture is a necessary way to synergistically exert the strengthening effect of different types of architectures. To realize the reasonable design of multitype composite architecture, it is very important to use simulation technology for the restructuring and mechanical analysis of multitype architectures. Besides, machine learning technology can be used to analyze and select the types and reorganization methods of architectures, which is helpful to accurately and reasonably design composite materials with excellent performance. Furthermore, we expect that the combination of various architectures can not only improve the mechanical properties but also produce some new functions. Taking the honeycomb material as an example, arranging CNTs into a honeycomb structure can achieve high conductivity and photothermal properties. In addition, the reticular hydrogel can be processed into honeycombs for cultivating living cells. Therefore, we predict that the combination of multiple architectures will be the future development trend. Understanding the design principle of each architecture and mastering computer‐aided technology (mechanical analysis and 3D modeling) are crucial for designing multitype composite architectures.

Third, structural coupling is the key to improving the performance of composites. The strengthening mechanisms of different structures are different. The general principle of structural coupling is to hide structural defects and enhance structural advantages. Therefore, understanding the strengthening mechanism of each architecture and designing the proper structure are essential to improve performance. For the coupling of different architectures, the problem to be solved is the conflict between functions that may occur in the design. Simulation is a feasible strategy. Through the simulation of the structure, we can understand its influence on mechanics, conductivity, magnetism, sound, and catalysis. Machine learning and theoretical prediction are other feasible strategies. Artificial intelligence can help structural design and function prediction by processing a large number of data sets.

Fourth, sophisticated preparation technology is a necessary condition to support the above viewpoint. Although some preparation technologies have been developed maturely, complex architectures require higher precision and a variety of raw materials. At the same time, the preparation technology of multiscale architecture should be developed to achieve the processing of hierarchical structures in actual production. The improvement of the preparation method can be started with processing accuracy and assembly technology. The dimensional accuracy of the reinforcement can be achieved by electron beam cutting, chemical etching, and 3D printing. Assembly technology needs to ensure that the structure of reinforcement is not damaged and polluted. Besides, the scalability of the preparation method should also be considered. The preparation method of composites shall meet the requirements of industrial practice.

Fifth, advanced characterization technology (such as scanning electron microscope, transmission electron microscope, X‐ray diffraction, nuclear magnetic resonance, mass spectrum, differential scanning calorimetry, tensile test, and hardness test) is conducive to microscopic morphology characterization, phase analysis, composition analysis, thermal property analysis, and mechanical property testing. These tools further help us understand the relationships among architecture, performance, and function. The characterization of structural materials can guide us back into the design and optimization of the structure and further improve the performance and function of composite materials. For structural materials, we suggest that characterization technology should achieve full 3D scanning of nanostructures and observe the dynamic evolution of physical and chemical responses in the future.

Sixth, the application of bionic structural composites will be more extensive in the future. In the field of national defense and industry, the improvement of strength and plasticity of metal matrix composites and ceramic matrix composites is still a challenge. It has great potential to improve strength and plasticity through structural design. In the field of medicine, bionic scaffolds, artificial bones, drug‐release materials, and living cell culture dishes all need structural design. Even in daily life, structural material with multiple functions can be seen everywhere. Although structural composites have developed rapidly, the preparation and processing technology still cannot keep up with the pace of development. Therefore, future research should not only focus on the preparation of composites, but also on their machinability. We believe that with the development of computer technology and manufacturing technology, more and more excellent composite materials will be developed.

In view of these aspects, we hope that through the comprehensive introduction of architecture, researchers will have a deeper understanding and inspiration in this field, and promote the development of bionic structural materials from the single‐architecture to multitype composite architecture. We believe that with the progress of preparation technology and design methods, the development of multitype composite architectures will enter a stable rising period.

## Conflict of Interest

The authors declare no conflict of interest.
